# Identification of the Carcinogenic Process from Lobular Endocervical Glandular Hyperplasia to Gastric-Type Adenocarcinoma of the Uterine Cervix via Whole-Exome Sequencing

**DOI:** 10.3390/cancers18040651

**Published:** 2026-02-17

**Authors:** Airi Kuruma, Tatsuo Masuda, Kazuaki Sato, Kansuke Kido, Daisuke Motooka, Naoko Komura, Takeshi Yokoi, Kosuke Yoshihara, Yasuto Kinose, Kae Hashimoto, Kenjiro Sawada, Eiichi Morii, Tadashi Kimura, Michiko Kodama

**Affiliations:** 1 Department of Obstetrics and Gynecology, Graduate School of Medicine, The University of Osaka, 2-2 Yamadaoka, Suita, Osaka 565-0871, Japan; 2 Osaka Regional Center for Japan Environment and Children’s Study (JECS), The University of Osaka, 2-2 Yamadaoka, Suita, Osaka 565-0871, Japan; 3 Department of Pathology, Graduate School of Medicine, The University of Osaka, 2-2 Yamadaoka, Suita, Osaka 565-0871, Japan; 4 NGS Core Facility, Research Institute for Microbial Diseases, The University of Osaka, 3-1 Yamadaoka, Suita, Osaka 565-0871, Japan; 5 BIKEN-RIMD NGS Laboratory, Research Institute for Microbial Diseases, The University of Osaka, 3-1 Yamadaoka, Suita, Osaka 565-0871, Japan; 6 Department of Obstetrics and Gynecology, Kaizuka City Hospital, 3-10-20 Hori, Kaizuka, Osaka 597-0015, Japan; 7 Department of Obstetrics and Gynecology, Graduate School of Medical and Dental Sciences, Niigata University, 1-757 Asahimachi-dori, Chuo-ku, Niigata-shi, Niigata 951-8510, Japan

**Keywords:** gastric-type adenocarcinoma, lobular endocervical glandular hyperplasia, whole-exome sequencing, clonal evolution, precursor lesion

## Abstract

**Background**: Gastric-type adenocarcinoma (GAS) of the uterine cervix is a rare malignancy with poor clinical outcomes. However, the carcinogenic processes involved remain unclear. **Methods**: Normal cervical glands, lobular endocervical glandular hyperplasia (LEGH), and GAS from the same patients were collected using laser microdissection for whole-exome sequencing. Single nucleotide variants (SNVs) and copy number alterations (CNAs) were analyzed. Phylogenetic trees were constructed based on the SNV and CNA profiles. **Results**: Analysis of seven matched samples demonstrated higher frequency of somatic mutations in the exonic regions in GAS than in LEGH. CNAs were prevalent in GAS but rare in LEGH. The phylogenetic analyses revealed various branching patterns. However, in three cases, the data suggested a sequential transition from LEGH to GAS, potentially associated with mutations in receptor-type protein tyrosine phosphatases such as *PTPRF* and *PTPRT*. *STK11* and *ARID1A* mutations were present in LEGH, with an increased variant allele frequency observed in GAS. In contrast, *SMAD4* and *SMAD2* showed frequent loss-of-function–type alterations in GAS, including copy-number loss, but were not detected in LEGH. **Conclusions**: These findings provide insights into the genomic landscapes of LEGH and GAS and suggest potential molecular markers for this transition, which may inform future diagnostic and therapeutic research.

## Simple Summary

Gastric-type adenocarcinoma of the uterine cervix is a rare but aggressive cancer that is difficult to detect early and often has a poor prognosis. Lobular endocervical glandular hyperplasia is considered a possible precursor lesion, but the genetic relationship between these lesions has not been fully clarified. In this study, we analyzed tissue samples of normal cervical glands, lobular endocervical glandular hyperplasia, and gastric-type adenocarcinoma obtained from the same patients using whole-exome sequencing. By comparing genetic alterations across these lesions, we found that some cases showed evidence supporting a sequential transition from lobular endocervical glandular hyperplasia to gastric-type adenocarcinoma, whereas other cases appeared to develop through alternative pathways. We also identified candidate genetic changes that may occur at early and late stages of tumor development. These findings provide new insights into the heterogeneous carcinogenic processes of this rare cervical cancer and may contribute to improved understanding of its development and inform future research on diagnosis and treatment.

## 1. Background

Gastric-type adenocarcinoma (GAS) of the uterine cervix is a rare subtype of endocervical adenocarcinoma (AC) that is independent of human papillomavirus (HPV) infection. In Japan, GAS accounts for approximately 10–20% of AC cases and is therefore not considered uncommon [1]. The diagnosis of GAS is often delayed because cytological screening is less effective for AC than for squamous cell carcinoma (SCC), and HPV testing is ineffective owing to the HPV-independent characteristics of GAS [2,3]. In addition, GAS may remain invisible at the mucosal surface even when invasive. Routine colposcopic biopsies, which are typically superficial, often fail to provide diagnostic tissue [4]. Consequently, GAS is often diagnosed at an advanced stage [5]. Moreover, GAS exhibits aggressive behavior, extensive involvement, and resistance to standard radiotherapy and chemotherapy, resulting in poor clinical outcomes [6,7]. Therefore, novel therapeutic strategies are urgently needed.

Several studies have described the pathologic and molecular characteristics of lobular endocervical glandular hyperplasia (LEGH), which is a benign lesion closely resembling the pathological findings of GAS. Morphologically, LEGH exhibits expanded glandular structures with medium- to small-sized glandular branching, similar to pyloric glands. These structures display circular nuclei without atypia and abundant cytoplasmic mucin, features consistent with gastric-type differentiation [8]. Immunohistochemically, gastric-type mucin markers such as HIK1083 and MUC6 are frequently positive. GAS also shows morphological similarities with gastric and pancreatic ductal adenocarcinoma (PDAC). HIK1083 and MUC6 are frequently positive in approximately 70% of GAS cases, p53 is positive in 50%, and p16 is usually negative or sporadically positive owing to HPV-independent features [9,10,11]. Considering the morphological and immunohistochemical similarities between LEGH and GAS, as well as their spatial coexistence, LEGH is considered a potential precursor lesion of GAS.

Recent targeted sequencing studies identified genetic mutations associated with LEGH and GAS. Among these reports using targeted sequencing, *TP53* mutation was the most frequent in GAS (25.0–53.3%), followed by mutations in *CDKN2A*, *KRAS*, *SLX4*, *STK11*, *ARID1A*, *BRCA2*, *SMAD4*, *ERBB2*, *GNAS*, and *ERBB3* [12,13,14,15,16,17]. Importantly, LEGH shares some of these mutation profiles, including *GNAS*, *KRAS*, and *STK11* [18], further supporting its putative role as a precursor lesion. However, molecular mechanisms underlying the transition from LEGH to GAS remain unclear. In this study, we performed whole-exome sequencing (WES) of paired samples of normal cervical gland, LEGH, and GAS collected by laser microdissection (LMD). We analyzed the somatic variants, including single nucleotide variants (SNVs), insertions and deletions (indels), and copy number alterations (CNAs). Additionally, mutation signature and phylogenetic analyses were performed to characterize genomic transitions and identify novel candidate genes related to carcinogenesis of GAS.

## 2. Methods

### 2.1. Study Participants and Tissue Samples

Formalin-fixed, paraffin-embedded (FFPE) blocks were obtained from seven patients histopathologically diagnosed with coexisting GAS and LEGH at the University of Osaka Hospital and Kaizuka City Hospital between August 2017 and September 2021. Clinical data, including age, medical history, clinical symptoms, treatment, tumor stage according to the International Federation of Gynecology and Obstetrics (FIGO) 2018, type of treatment, and prognosis, were extracted from medical records. A gynecologic oncology pathologist re-evaluated the pathological images and confirmed the diagnosis of LEGH and GAS based on the World Health Organization 2020 classification of cervical cancer. This study was approved by the Institutional Review Board of the University of Osaka (IRB no.22040-6), and informed consent was obtained from the website.

### 2.2. LMD

Five to ten serial sections, the thickness of which was 14 μm for LMD (Leica LMD7, Wetzlar, Germany) and was 4 μm for hematoxylin and eosin (HE) staining, were obtained from FFPE blocks using a rotary microtome. HE staining of serial sections was reviewed by a gynecologic pathologist to identify areas in which normal cervical glands, LEGH, and GAS could be clearly diagnosed. Gastric-type adenocarcinoma in situ (GAIS) and atypical LEGH areas were excluded because it was difficult to precisely distinguish them from GAS and LEGH areas during LMD. Only the gland duct structures were collected from the normal cervical gland, LEGH, and GAS. Normal myometrium was collected from all patients as a reference to exclude germline variants.

### 2.3. DNA Extraction

Genomic DNAs were extracted using QIAamp DNA FFPE Advances UCG kits (Qiagen, Hilden, Germany), according to the manufacturer’s protocols. DNA purity was assessed using NanoDrop 2000 (Thermo Fisher, Waltham, MA, USA).

### 2.4. Immunohistochemistry Staining

p53 immunohistochemistry (IHC) was performed using an automated stainer (VENTANA BenchMark ULTRA, Roche Diagnostics K.K., Tokyo, Japan) with clone D07 antibody (cat. no. 05278074001, Roche, Basel, Switzerland)

### 2.5. Library Construction, Target Capturing, and WES

Library construction and exome capture were performed using Sure SelectXT Low Input Reagent Kits (Agilent, Santa Clara, CA, USA) and SureSelect Human All Exon v6 kit (Agilentt, Santa Clara, CA, USA). The captured DNA was sequenced using a DNBSEQ-G400RS (MGI-Tech, Shenzhen, Guangdong, China) to generate 100-bp paired-end reads.

### 2.6. Quality Control, Variant Calling, and Somatic Short Variant Analysis

Raw sequence reads were aligned to the GRCh38 human reference genome (Broad Institute, Cambridge, MA, USA) using the Burrows–Wheeler Aligner (BWA, version 0.7.15). Binary Alignment/Map (BAM) files were analyzed for somatic mutations and CNAs following the Best Practice Workflow of the Genome Analysis Toolkit (GATK, version 4.2.6.1; Broad Institute, Cambridge, MA, USA) [19]. PCR duplicates were removed, followed by local realignment and base-quality recalibration. SNVs and indels were called using Mutect2 implemented in GATK version 4.2.6.1 [20,21]. Hereafter, SNVs and indels will be referred to as short variants. Variants present in normal myometrium were excluded from the paired samples as germline variants. Low-quality variants were filtered for mean base quality, depth, and variant allele frequency (VAF) using SnpEff (version 4.3p) [22]. The identified variants were annotated using ANNOVAR (2017jun08; Wang Genomics Lab, University of Pennsylvania, Philadelphia, PA, USA). Mutations in unknown chromosomal locations or mitochondrial chromosomes were excluded. Mutation signature analysis of SNVs was performed using the R (version 4.4.1; R Foundation for Statistical Computing, Vienna, Austria) using the package MutSignature (version 2.1.5).

### 2.7. CNA Analysis

CNAs were identified using the somatic copy number variant discovery protocol in GATK. To detect CNAs where at least 20% of tumor cells harbored gain or loss of one of the two alleles, cutoff values of copy ratios for copy number gain and loss were set at >1.1 and <0.9, respectively. Copy-neutral loss of heterozygosity (CN-LOH) was defined as no total copy number change (copy ratio between 0.9 and 1.1) but with a deviated minor allele fraction < 44%. The cut-off value for the minor allele fraction was determined as the mean of −3 standard deviations for the 10% tile values of the minor allele fraction among the seven normal myometrium samples. Haplotype phasing was performed using Beagle software (version 5.4) to assess which haplotype harbored the short variants and segments of the CNAs.

### 2.8. Phylogenetic Analysis of Short Variants and CNAs

Phylogenies of the short variants for each patient were reconstructed using the DNA parsimony algorithm in the PHYLIP program (version 3.697). The trees in the Newick format were rendered using the R package ggplot2 (version 3.4.4). To assess robustness, we additionally reconstructed maximum-likelihood phylogenetic trees using phangorn (version 2.12.1) and ape (version 5.8-1). Phylogenetic trees of the CNAs were modeled using MEDICC2 (version 1.0.2).

### 2.9. Scoring System for Identifying Candidate GAS-Related Genes

The known cancer genes in the Cancer Gene Census (CGC) database (https://cancer.sanger.ac.uk/census, accessed on 23 November 2023) were matched to the genes for which short variants and/or CNAs were detected in the GAS. To stratify the candidate genes, the scoring method was defined as follows: First, we considered exonic short variants (excluding synonymous SNVs) that had a minor allele frequency of less than 0.01 or unregistered variants in the East Asian population of the gnomAD database (i.e., likely to be somatic mutations). If such a mutation was detected in one case, one point was assigned; if it was detected in two cases, two points were assigned. Even if multiple mutations were present in the same gene within the same case, only one point was assigned to the gene (to avoid double counting). Second, one point was assigned to the gene if it was involved in a biologically plausible copy number event (for example, copy number gain of oncogenes or loss of tumor suppressor genes) according to the CGC database. CN-LOH was considered only when functional consequences could be inferred. For example, CN-LOH was only scored when a loss-of-function variant was located in the retained (gained) haplotype. CN-LOH events were not scored if the function of the associated variants was unclear. Points were conferred to the genes based on the number of cases with CNA that met the requirements. The final gene score was calculated by summing the points from the short variants and CNA evaluations. Genes with two or more points were classified as candidate GAS-related genes. Data visualization was performed using R package ComplexHeatmap (version 2.14.0). Pathway analysis was performed using DAVID (The Database for Annotation, Visualization and Integrated Discovery) tool for genes that scored three or more points [23]. False discovery rate by Benjamini–Hochberg method less than 0.05 was considered statistically significant. We performed a threshold sensitivity analysis using stricter cutoffs (≥4 and ≥5) (Supplementary Table S7).

### 2.10. Statistical Analysis

The Kruskal–Wallis test was used to compare continuous variables among the three groups, and the Wilcoxon rank-sum test was performed to compare continuous variables between two groups. A *p*-value of <0.05 was considered statistically significant. When significant in the Kruskal–Wallis analysis, the Wilcoxon rank-sum test was performed to compare pairs of groups. In such cases, corrected *p*-values for multiple testing were used. All statistical analyses were performed using JMP Pro version 17.1.0 (SAS Institute Inc., Cary, NC, USA).

## 3. Results

### 3.1. Patients’ Clinical Characteristics

**Figure 1:**
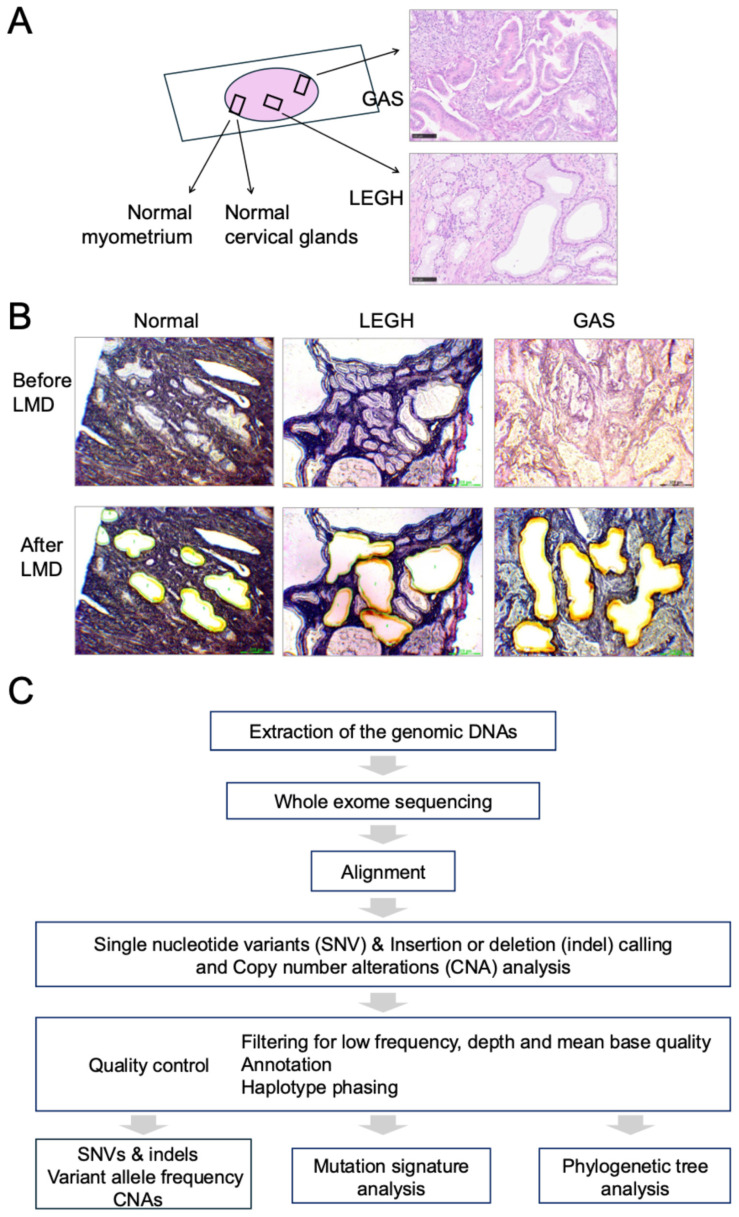
Overview of the study design, sample preparation, and bioinformatics workflow. (**A**) Representative schema of sample selection. In each case, normal myometrium, normal cervical glands, lobular endocervical glandular hyperplasia (LEGH), and gastric-type adenocarcinoma (GAS) were identified. Hematoxylin and eosin-stained images of LEGH and GAS are shown. Original magnification, ×200. Scale bar = 100 μm. (**B**) Representative images of laser microdissection. Images before (upper panel) and after (lower panel) laser capture microdissection are shown. (**C**) Whole-exome-sequencing workflow and bioinformatics pipeline used in this study.

Seven patients were included in this analysis. The clinical characteristics of the patients are summarized in Table 1. The median age at diagnosis was 47 years (range, 32–84). One patient had a history of benign small intestinal tumors, and another patient had a history of both breast and thyroid cancers. None of the patients had any history of hereditary cancer. According to FIGO 2018 classification, the clinical stages were as follows: three cases of IB1, one case of IB2, one case of IIA2, one case of IIB, and one case of IIIA. All specimens, including normal cervical glands, LEGH, and GAS, were obtained from the same patient during conization or hysterectomy prior to radiation or chemotherapy. HE staining and p53 IHC for each case are shown in Figure 1A and Supplementary Figure S1. At the time of this analysis, one patient had persistent disease, another had recurrent disease and the remaining five showed no evidence of disease.

### 3.2. WES Summary

Genomic DNA was extracted from normal myometrium, normal cervical glands, LEGH, and GAS obtained from each patient using LMD (Figure 1A,B). The mean DNA concentration from all samples was 40.4 ng/μL (Supplementary Table S1). DNA extraction from the normal cervical glands in Cases 1 and 6 was not performed because of the small amount of tissue. In total, 26 matched samples from seven cases, including the myometrium, normal cervical glands, LEGH, and GAS, were analyzed using WES. The total read counts were 100,295,382 ± 36,342,263 (mean ± standard deviation), with a coverage ratio of 0.21 ± 0.06, and a depth of coverage of 15.9 ± 3.9 (Supplementary Table S1).

### 3.3. Somatic Mutation Analysis

**Figure 2:**
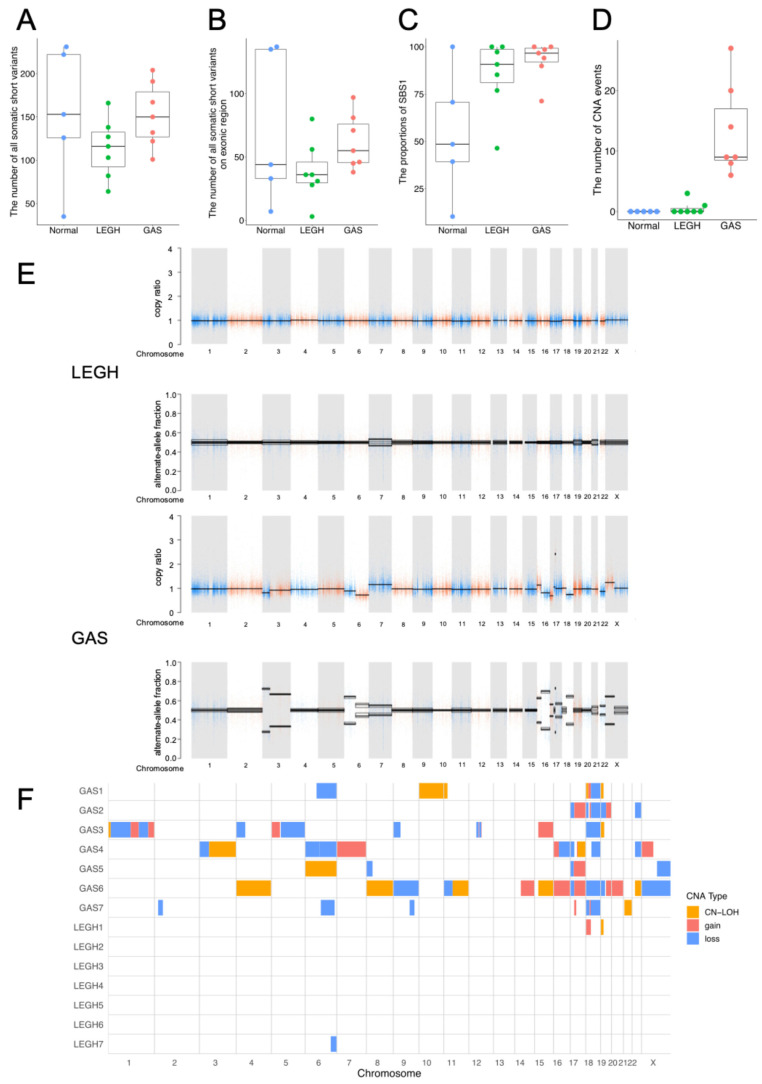
Summary of somatic alterations of normal cervical glands, LEGH, and GAS. (**A**) Total number of somatic short variants detected in each sample—normal cervical glands (Normal, *n* = 5), LEGH (*n* = 7), and GAS (*n* = 7). Boxplots show the median, interquartile range, and range. Colors represent Normal (blue), LEGH (green), and GAS (red). (**B**) The number of exonic mutations per sample is shown in boxplots. (**C**) The proportions of SBS1 are compared among tissues. There were no significant differences by Kruskal–Wallis test. The mean rank scores in Wilcoxon rank sum test were 5.8 (Normal), 10.7 (LEGH), and 12.3 (GAS). A low proportion tended to be observed in normal glands while a high proportion was observed in GAS. (**D**) The number of CNA events per sample is shown. No CNAs were detected in normal glands. Significant differences were observed among groups (*p* < 0.01, Kruskal–Wallis test). GAS showed significantly more CNA events than Normal and LEGH (*p* < 0.02, corrected *p*-values for multiple comparisons). (**E**) Representative image of CNA calling results, illustrated by LEGH and GAS in Case 4. Results were obtained using the somatic copy number variant discovery protocol in Genome Analysis Toolkit (GATK). The *x*-axis indicates the chromosomal positions, and the *y*-axis represents the copy ratio or alternate allele fraction. (**F**) CNA profiles of LEGH and GAS samples are shown. Red indicates copy number gain, light blue indicates copy number loss, and yellow indicates CN-LOH. Chromosomal positions are shown on the *x*-axis, and sample names (e.g., GAS1 = GAS sample from Case 1) are shown on the *y*-axis. Abbreviations: LEGH, lobular endocervical glandular hyperplasia; GAS, gastric-type adenocarcinoma; SBS1, single-base substitution signature 1; CNA, copy number alteration; CN-LOH, copy-neutral loss of heterozygosity.

The WES workflow and bioinformatics pipeline are shown in Figure 1C. The median number of somatic mutations was 153 (range: 35–231) in normal cervical glands, 116 (64–166) in LEGH, and 150 (101–204) in GAS (Figure 2A and Supplementary Table S1). In exonic regions, the median number of somatic mutations was 36 (range: 3–80) in LEGH and 55 (range: 38–97) in GAS, indicating a higher somatic mutation burden in GAS than in LEGH (Figure 2B and Supplementary Table S1). The mean mutation rate was 0.21 ± 0.08/Mb across all cases, including 0.24 ± 0.11/Mb in normal cervical glands, 0.17 ± 0.07/Mb in LEGH, and 0.22 ± 0.06/Mb in GAS (Supplementary Table S1). Frameshift, stop-gain, and nonsynonymous SNVs were more frequent in GAS than in LEGH (Supplementary Figure S2 and Supplementary Table S2).

### 3.4. Mutational Signature Analysis

To investigate the biological processes and background factors contributing to mutations in LEGH and GAS, a mutational signature analysis was performed. Signature analysis of SNVs revealed that single-base substitution (SBS) 1, a clock-like signature associated with aging, was the predominant mutational pattern. Other SBS signatures were also detected, although less frequently, in normal glands and LEGH. A trend toward increasing SBS1 proportions was observed from normal glands to LEGH and GAS, although the difference was not statistically significant (Figure 2C). In contrast, SBS2 and SBS13, which are commonly observed in HPV-driven cervical cancers, were not represented in this study (Supplementary Figure S3 and Supplementary Table S3).

### 3.5. CNA Landscape

CNA events were predominantly detected in GAS but were very rare in LEGH (Figure 2D,E and Supplementary Figure S4). Three mutual CNA events were identified between LEGH and GAS in two cases: loss of chromosome (chr) 6q in Case 7 and gain of chr 18p and CN-LOH of chr 19p in Case 1 (Figure 2F). These findings supported the hypothesis of a sequential transition from LEGH to GAS. Frequent CNAs observed in GAS included copy number loss or CN-LOH of chr 6q, gain of chr 17 and chr 18p, and CN-LOH or loss of chr 19p. However, CNAs involving other chromosomal regions were nonspecific in GAS (Figure 2F).

### 3.6. Assessment of the Known GAS-Related Genes in LEGH and GAS

**Figure 3:**
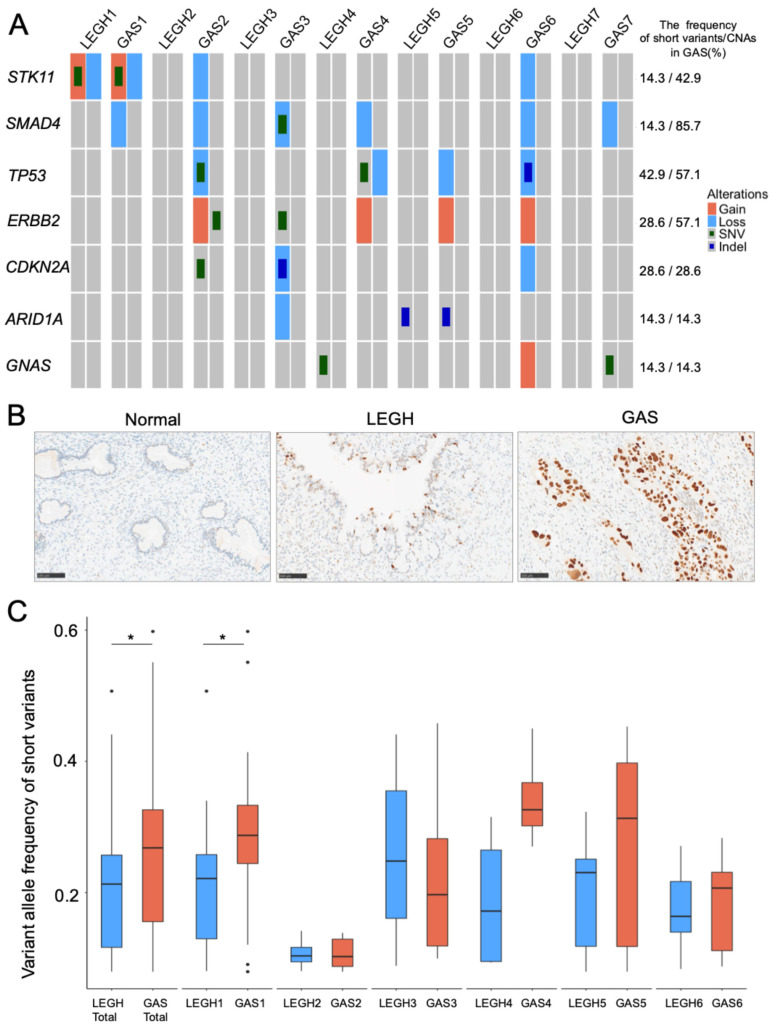
Genetic alterations in representative GAS-related genes in LEGH and GAS. (**A**) Detected short variants and CNAs in representative known GAS-related genes are displayed. Each pair of gray boxes represents the two haplotypes of each gene. Red and blue represent copy number gain and loss, respectively. Green small squares and dark blue small squares represent SNV and short insertions/deletions, respectively. Numbers on the right indicate the frequency of each short variant and CNA, respectively. (**B**) Representative images of p53 immunostaining in Case 2 are shown. p53 expression is almost negative in normal glands (Normal) (**left**), shows partial nuclear staining with varying intensity from weak to strong in LEGH (**center**), and diffuse and strong nuclear staining in GAS (**right**). (**C**) VAFs of mutual short variants commonly detected in both LEGH and GAS are shown. “Total” refers to the mean VAF of all these cases. LEGH1 refers to LEGH in Case 1. There were 133 mutually mutated genes in all cases: 70 in LEGH1 and GAS1, 7 in LEGH2 and GAS2, 17 in LEGH3 and GAS3, 4 in LEGH4 and GAS4, 18 in LEGH5 and GAS5, and 17 in LEGH6 and GAS6. Boxplots represent the median and interquartile ranges (blue: LEGH, red: GAS). Asterisks indicate *p* < 0.01. Abbreviations: CNA, copy number alteration; LEGH, lobular endocervical glandular hyperplasia; GAS, gastric-type adenocarcinoma; SNV, single nucleotide variant; VAF, variant allele frequency.

Previously reported GAS-related genes in LEGH and GAS were assessed using our dataset (Figure 3A). Short variants were detected in *TP53* (3/7, 42.9%), *CDKN2A* (2/7, 28.6%), *ERBB2* (2/7, 28.6%), *STK11*, *ARID1A*, *SMAD4*, and *GNAS* (1/7, 14.3%). The regions of copy number loss included *SMAD4* (6/7, 85.7%), *TP53* (4/7, 57.1%), and *CDKN2A* (2/7, 28.6%), while the region with copy number gain covered *ERBB2* (4/7, 57.1%) gene in GAS. IHC results for p53 suggested functional gain in Cases 2 and 5, functional loss in Case 6, and the absence of mutations in Case 4 (Figure 3B and Supplementary Figure S1). However, WES revealed loss of the haplotype harboring the mutated *TP53* in Cases 2 and 6 and loss of the haplotype harboring normal *TP53* in Cases 4 and 5. Somatic mutations in *STK11*, *ARID1A*, and *GNAS* have been identified in LEGH. Mutations in *STK11* in Case 1 and *ARID1A* in Case 5 were observed in both GAS and LEGH (Figure 3A). These mutations showed increased VAFs in GAS compared to those in LEGH, particularly in *STK11* mutation, where the VAF was already high in LEGH (0.50 for LEGH and 0.59 for GAS) (Supplementary Figure S5). Further analysis using haplotype phasing revealed that the copy number of the haplotype containing mutated *STK11* was gained, while the copy number of the haplotype containing normal *STK11* was lost (Figure 3A). In addition, hotspot mutations in *GNAS* were identified in LEGH of Case 4 and GAS of Case 7 (Figure 3A and Supplementary Table S4). For each case, we examined the VAF of all mutations between LEGH and GAS, including those in non-exonic regions. A total of 133 mutual mutations were identified in all cases except Case 7. Although the tumor regions isolated by LMD may not fully represent the entire tumor, the mean VAF of shared mutations was modestly higher in GAS than in LEGH (0.26 vs. 0.20, *p* < 0.01; Figure 3C). This pattern was observed in most cases, with the exception of Cases 2 and 3 (Figure 3C and Supplementary Figure S5).

### 3.7. Phylogenetic Analysis

**Figure 4:**
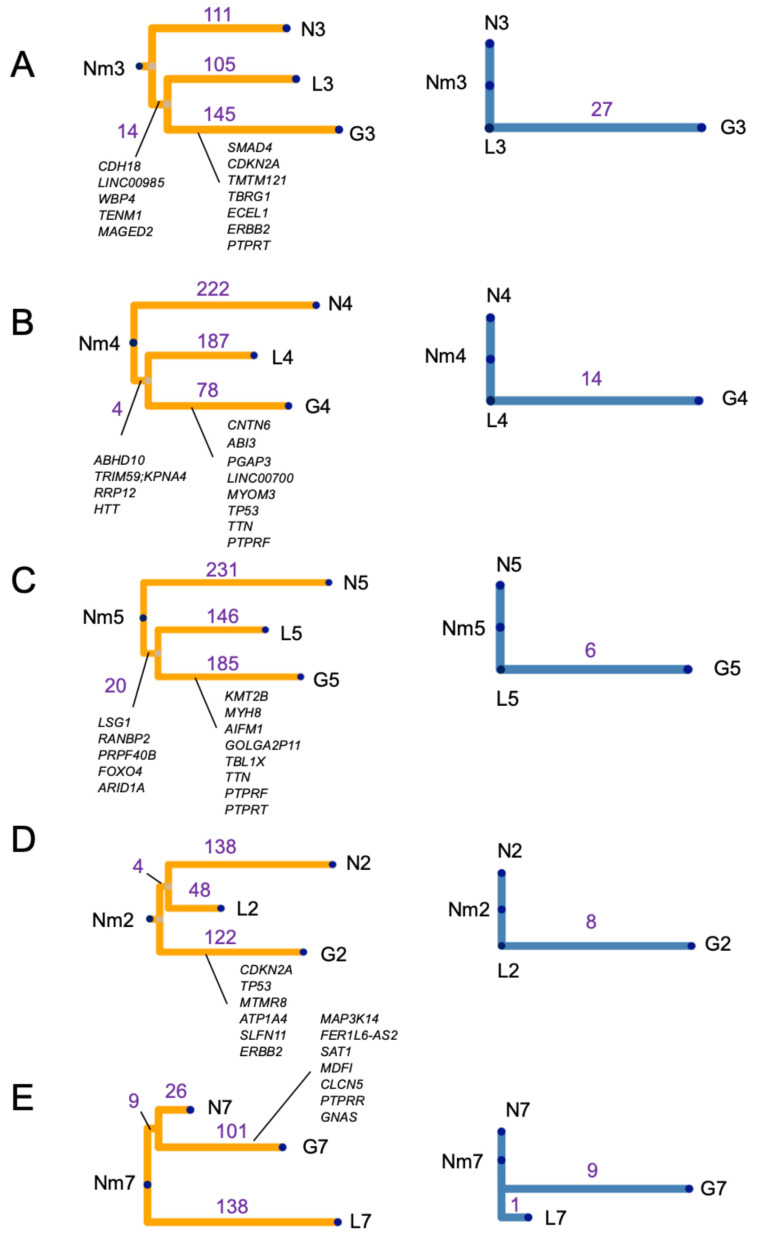
Phylogenetic analysis of short variants and CNAs in LEGH and GAS. (**A**–**E**) Phylogenetic trees from short variants (yellow) and CNAs (blue). (**A**) Case 3, (**B**) Case 4, (**C**) Case 5, (**D**) Case 2, and (**E**) Case 7. Numbers along each branch (purple) indicate the number of short variants or CNA events. Among the mutations detected in GAS, the five genes with the highest VAFs, genes commonly mutated in GAS in at least two of the three cases (Cases 3, 4, and 5), and representative known GAS-related genes are listed. Mutual mutations between LEGH and GAS are ordered by descending VAFs of GAS. Abbreviations: CNA, copy number alteration; VAF, variant allele frequency; LEGH, lobular endocervical glandular hyperplasia; GAS, gastric-type adenocarcinoma; Nm, normal myometrium; N, normal cervical gland; L, LEGH; G, GAS.

Phylogenetic trees were evaluated in five cases, excluding Cases 1 and 6 owing to the absence of normal cervical gland data (Supplementary Figure S6). In these two cases lacking matched normal cervical gland specimens, phylogenetic reconstruction between LEGH and GAS was still performed; however, inference of evolutionary directionality was limited without a normal baseline. Three distinct phylogenetic tree patterns constructed from the short variants were identified (Figure 4A–E). In Type 1 pattern, LEGH and GAS shared a common branch (Figure 4A–C), suggesting a transition from LEGH to GAS. Three of the five cases exhibited this Type 1 pattern. *PTPRF* and *PTPRT* were commonly mutated in GAS (Supplementary Figure S6C). Mutual mutations in the common branches were arranged in the order of increasing VAF in GAS. In Type 2, a common branch was shared between normal cervical glands and LEGH, but not between LEGH and GAS (Figure 4D). In Type 3, a common branch was shared between normal glands and GAS, but not between LEGH and GAS (Figure 4E). In Type 2 and 3, the phylogenetic relationship between LEGH and GAS could not be conclusively resolved from the available sampling, indicating heterogeneity across cases. Maximum-likelihood reconstruction yielded an overall concordant tree topology with the primary analysis. The phylogenetic trees of the CNAs were consistent with those of the short variants, suggesting that CNA accumulation contributes to GAS carcinogenesis (Figure 2D and Figure 4A–E).

### 3.8. Investigation of Novel Alterations in GAS

**Figure 5:**
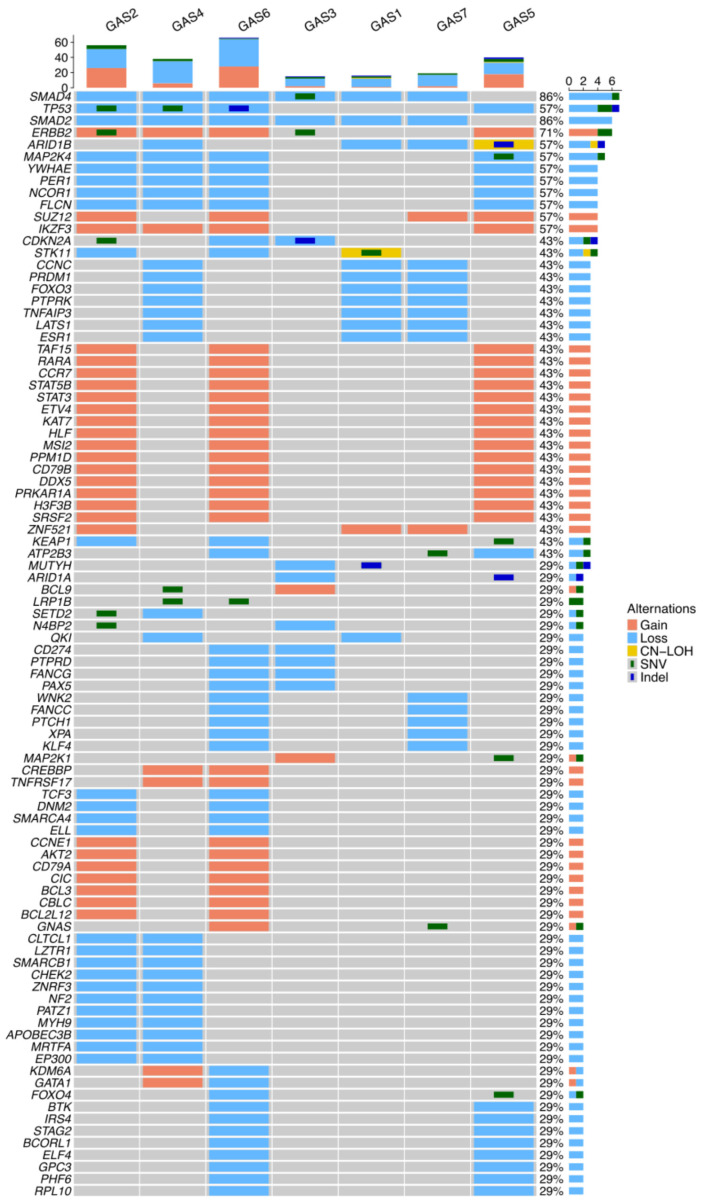
Prioritized GAS-related gene candidates and their associated pathways. Heatmap of the top 92 candidate genes that scored at least two points based on our mutation scoring system are shown. Red indicates copy number gain, light blue indicates copy number loss, yellow indicates copy-neutral loss of heterozygosity (CN-LOH), green square indicates single nucleotide variants (SNVs), and dark blue square indicates short insertions or deletions (indels). Gray indicates no detected alteration. The percentage values on the right represent the proportion of cases with alterations in each gene among the seven GAS samples. The bar plot on the right shows the distribution of mutation types across genes. Rows and columns were automatically clustered. The bar plot at the top represents the composition of alteration types in each case. Abbreviations: GAS, gastric-type adenocarcinoma.

Next, we ranked known cancer-related genes based on the short variants and/or the CNAs identified in our dataset. Of the 743 genes listed in the CGC database, short variants or CNAs were detected in 652 genes. Based on our scoring system, 257 genes scored at least one point (Supplementary Table S5), and 92 genes that scored two or more points were identified as candidate GAS-related genes (Figure 5. Cancer-related genes, such as *SMAD4*, *SMAD2*, *TP53*, *ERBB2*, *ARID1B*, and *MAP2K4*, were ranked at the top. A threshold sensitivity analysis retained a stable subset of top-ranked genes under stricter cutoffs (≥4 and ≥5) (Supplementary Table S7). Pathway enrichment analysis was performed using the 39 genes that scored three or more points. This exploratory analysis identified enrichment of Kyoto Encyclopedia of Genes and Genomes (KEGG) cancer-related pathways, including the non-small cell lung cancer pathway, pancreatic cancer pathway, pathways in cancer, and hepatocellular carcinoma pathway (Supplementary Table S6).

## 4. Discussion

In this study, we provide genomic evidence consistent with stepwise progression from LEGH to GAS based on paired clinical samples and comprehensive WES. Multiple lines of genomic evidence—including shared mutations, phylogenetic relationships based on short variants and CNAs, mutational signatures, and VAF patterns—were collectively consistent with this model.

Mutational signature analysis was broadly consistent with an endogenous mutational process. SBS1, a clock-like signature, was predominant across lesions, and its proportion showed a non-significant trend toward increase from normal glands to LEGH and GAS. Importantly, APOBEC-associated signatures (SBS2 and SBS13), which are commonly observed in HPV-driven cervical carcinogenesis, were not detected, consistent with an HPV-independent mutational landscape in GAS.

Despite the limited sample size, the observed evolutionary trajectories across this cohort were not uniform, suggesting that subclonal expansion patterns vary among individual tumors. In Type 2 and 3 patterns, the precise phylogenetic relationship between LEGH and GAS unresolved with the current sampling. The variety in VAF dynamics, including instances where VAFs were higher in LEGH than in GAS, highlights the profound complexity of clonal evolution, likely influenced by spatial intratumoral heterogeneity and sampling bias. Taken together, these findings do not negate stepwise progression but rather suggest that gastric-type cervical carcinogenesis may involve multiple evolutionary routes. These results warrant further validation in larger cohorts with broader sampling and spatially resolved genomic approaches. We further provided a comprehensive catalog of gene alterations associated with GAS development, including *SMAD4*, *SMAD2*, and other putative driver genes. Previous genomic studies on LEGH and GAS have utilized PCR-based assays or targeted sequencing and reported frequent mutations in GAS, including *TP53* (25.0–53.3%), *CDKN2A* (18.0–37.5%), *KRAS* (4.8–36%), *SLX4* (9.5–35.7%), *STK11* (10.0–33.3%), *ARID1A* (12.5–28.6%), *BRCA2* (9.5–21.4%), *SMAD4* (9.0–15.0%), *ERBB2* (8.0–13.3%), *GNAS* (9.0–13.3%), and *ERBB3* (9.0–10.0%). *GNAS*, *KRAS*, and *STK11* mutations have also been reported in LEGH, highlighting their role as precursor lesions [12,13,14,15,16,17,18]. Because these studies examined LEGH and GAS separately, they provided only limited insights into their direct relationships. In our cohort, the somatic mutation frequencies of GAS-related genes were generally consistent with those reported in previous studies, underscoring the reliability of our data. Among these genes, *TP53* expression was assessed by IHC; however, the IHC results were not always concordant with the genomic findings. Although p53 IHC is widely used as a surrogate marker for *TP53* alterations, discordance between immunostaining patterns and molecular status has been reported [24]. Several factors may contribute to this discrepancy. In addition, standard exome-based analyses may miss some variant classes relevant to gene regulation, and RNA/protein-level regulatory mechanisms may also affect protein abundance [25]. Notably, *STK11* and *ARID1A* mutations were detected in both LEGH and GAS, implying their role in early malignant transition. *STK11*, a tumor suppressor gene mutated in 10–33% of GAS, has been linked to the transition from LEGH to GAS [26] and poor prognosis [15]. In our study, a mutual SNV in *STK11* was detected in both LEGH and GAS, with a high VAF in LEGH, suggesting that *STK11* loss-of-function mutation may represent an early event in carcinogenesis. *ARID1A*, which encodes a subunit of the SWI/SNF (SWItch/Sucrose Non-Fermentable) complex, is frequently mutated in ovarian and endometrial cancers [27,28] and has also been reported in GAS [14]. To the best of our knowledge, this is the first report of an *ARID1A* mutation associated with LEGH. Similar to *ARID1A* inactivation, which drives early events during carcinogenesis in ovarian and endometrial cancers [28], its loss-of-function mutations (and/or copy number loss) may be related to early events in LEGH. Furthermore, alterations in other SWI/SNF subunit genes, such as *ARID1B* and *SMARCA4*, occurred in several cases of GAS but were absent in LEGH, implying their roles in the later stages of carcinogenesis, as previously reported in endometrial cancer [29].

Mutations in *PTPRF* and *PTPRT*, which are members of the receptor-type protein tyrosine phosphatase (PTPR) family, were commonly observed. PTPRs play key roles in regulating cell proliferation, differentiation, adhesion, and cell–cell communication in various cancers [30]. *PTPRS* mutations, one of the subfamilies of PTPRs, were detected in 19% of GAS [16]. *PTPRT* and *PTPRS* act as tumor suppressors, whereas *PTPRF* may function as either a suppressor or an oncogene depending on the context [30]. Another important finding of this study was the potential role of CNAs in GAS carcinogenesis. We observed frequent CNAs on chr 17, 18, and 19, involving genes such as *TP53*, *SMAD4*, *STK11*, and *PTPRS*, indicating their involvement not only through short variants but also through CNAs. Previous studies reported a gain of chr 3q and loss of chr 1p in LEGH and minimal deviation adenocarcinoma (MDA), a well-differentiated form of GAS, using comparative genomic hybridization [31], and loss of heterozygosity in 19p13.3 in MDA [32]. The discrepancies between these findings and ours may be due to differences in the sequencing methods. Notably, a recent study using parallel sequencing has reported CNA data similar to ours [17].

Among putative drivers, *SMAD4* and *SMAD2* were notable [17,33]. The *SMAD4* missense mutation, R361C, located in the MH2 domain, is a well-known hotspot in gastrointestinal cancers [34]. This domain is essential for homodimerization, heterodimerization, transcriptional activation, and nuclear localization of *SMAD4* [35]. Disruption of the MH2 domain is known to impair canonical TGF-β signaling, consistent with a loss-of-function mechanism and reduced tumor suppressor activity. In addition to coding mutations, the frequent copy number loss of *SMAD4* observed in our cohort suggests that gene dosage effects may contribute to functional impairment, even in the absence of detectable mutations. Notably, *SMAD2* is located in close proximity to *SMAD4* on chromosome 18q; therefore, *SMAD2* loss in GAS may partly reflect broader 18q loss events that also encompass *SMAD4*. Given that *SMAD2* and *SMAD4* are central mediators of canonical TGF-β signaling, loss-of-function alterations in these genes are biologically plausible and consistent with the tumor-suppressive role of this pathway in epithelial tissues. The predominance of such alterations in GAS rather than LEGH may reflect selective pressure during malignant progression, whereby disruption of growth-inhibitory signaling confers a proliferative advantage. Collectively, these findings support the notion that TGF-β pathway dysregulation may represent an important molecular event in GAS tumorigenesis. Similarly, alterations were found in *MAP2K4*, a kinase in the JNK/p38 MAPK pathway that functions as a tumor suppressor and has been implicated in breast, gastric, lung, pancreatic, and ovarian cancers [36,37,38,39,40]. Although the specific roles of *MAP2K4* in GAS remain unclear, the recurrent alterations observed here may have functional consequences. Further studies may pave the way for understanding the mechanism of tumorigenesis and possible approaches for intervention.

Our study had several limitations. First, the rarity of matched LEGH-GAS specimens limited the sample size, as most GAS cases were diagnosed at advanced stages when normal glandular structures were absent. Therefore, our findings should be considered exploratory and warrant validation in larger, independent cohorts. In addition, the gene prioritization score was intended as a heuristic tool. Although we assessed sensitivity to stricter score thresholds (≥4 and ≥5), we did not formally evaluate robustness under alternative weighting schemes. Moreover, phylogenetic reconstruction was feasible in only a subset of cases due to limited availability of matched normal glands, potentially affecting the interpretation of evolutionary trajectories. Second, bulk sequencing of laser-dissected lesions did not overcome the issue of tumor heterogeneity, although careful microscopic assessment likely minimized contamination. Third, the quality of the FFPE samples could not be guaranteed owing to degradation; however, our stringent quality control yielded valid and reliable results. Fourth, the analysis focused on localized early-stage tumors, limiting our ability to assess genes involved in progression and metastasis. However, this focus allowed us to investigate the early stages of carcinogenesis in detail. Fifth, functional validation of the putative driver genes was not performed; therefore, these findings should be interpreted as hypothesis-generating rather than definitive evidence of oncogenic function. Future in vitro and in vivo studies using primary models will be necessary to confirm their biological roles. Finally, the tumor microenvironment was not assessed because only epithelial components were analyzed, which may limit insight into tumor–stromal interactions and should be addressed in future studies using spatial transcriptomics.

## 5. Conclusions

In summary, our findings provide a potential comprehensive genomic landscape of sequential transition from LEGH to GAS. These insights advance our understanding of its molecular pathogenesis and may inform the development of early diagnostic biomarkers and precise intervention strategies.

## Figures and Tables

**Table 1:** Characteristics and clinical features of all patients.

Case	Age at Diagnosis	Medical History	Clinical Symptoms	FIGO Stage	The Type of Operation	Adjuvant Therapy	Prognosis
1	32	Condyloma	Abnormal discharge	IIIA	Conization	CCRT and chemotherapy	AWD
2	47	Heart disease	Abnormal cervical cytology	IB1	RH + BSO	CCRT	NED
3	47	Intestinal intussusception caused by small intestinal tumor	Abnormal discharge	IIA2	RH + BSO	CCRT	NED
4	47	None	Abnormal discharge	IB1	TLH + RSO	CCRT	NED
5	67	Breast cancer Thyroid cancer	Abnormal bleeding	IB1	SemiRH + BSO	CCRT	NED
6	53	Duodenal ulcer	Abnormal cervical cytology	IB2	Conization followed by RH	RT	NED
7	84	Gallstones	Hydrometra	IIB	RH	RT	AWD

Abbreviation: CCRT: concurrent chemoradiotherapy, RH: radical hysterectomy, BSO: bilateral salpingo-oophorectomy, TLH: total laparoscopic hysterectomy, RSO: right salpingo-oophorectomy, RT: radiotherapy, AWD: alive with disease, and NED: no evidence of disease.

## Data Availability

Raw sequencing data generated in this study are currently being prepared for deposition in the Japanese Genotype-phenotype Archive (JGA) under controlled access. Accession numbers will be available upon publication.

## Supplementary Materials

The following supporting information can be downloaded at: https://www.mdpi.com/article/10.3390/cancers18040651/s1, Supplementary Figure S1. Representative hematoxylin and eosin and p53 immunohistochemical (IHC) staining images of all cases. Magnification: ×200. Scale bar: 100 µm. Abbreviations: LEGH, lobular endocervical glandular hyperplasia; GAS, gastric-type adenocarcinoma. Supplementary Figure S2. The breakdown of exonic mutations of normal cervical glands (Normal), LEGH and GAS. Abbreviations: LEGH, lobular endocervical glandular hyperplasia; GAS, gastric-type adenocarcinoma; SNV, single nucleotide variant. Supplementary Figure S3. Mutational signatures. (A) A heatmap depicts the frequency of mutational signatures of all samples. Signatures other than SBS1 were detected in normal cervical glands (Normal) and LEGH, whereas SBS1 was more prevalent in GAS. High frequency is shown in dark blue, and low frequency is shown in pale blue. (B–H) Single-base substitution (SBS) profiles for each case. Abbreviations: SBS, single-base substitution; LEGH, lobular endocervical glandular hyperplasia; GAS, gastric-type adenocarcinoma. Supplementary Figure S4. Copy number alteration (CNA) calling results using the somatic copy number variant discovery protocol in Genome Analysis Toolkit (GATK). The x-axis indicates the chromosomal positions, and the y-axis represents the copy ratio or alternate allele fraction. Supplementary Figure S5. VAF profiles of mutual mutations in LEGH and GAS. The line graph shows VAF changes of mutual variants between LEGH and GAS. X-axis: sample type; Y-axis: VAF. Abbreviations: VAF, variant allele frequency; LEGH, lobular endocervical glandular hyperplasia; GAS, gastric-type adenocarcinoma. Supplementary Figure S6. Phylogenetic and mutational analysis of LEGH and GAS. (A) Phylogenetic trees based on short variants (yellow) and CNAs (blue) for Cases 1 and 6 are illustrated. Numbers on branches (purple) indicate mutation or CNA counts. (B) Newick format used for tree construction from short variants is shown. (C) Venn diagram of common mutations in Cases 3, 4, and 5, with shared genes indicated. Abbreviations: CNA, copy number alteration; SNV, single nucleotide variant; VAF, variant allele frequency; LEGH, lobular endocervical glandular hyperplasia; GAS, gastric-type adenocarcinoma; Nm, normal myometrium; N, normal; L, LEGH; G, GAS. Supplementary Table S1. Summary of all sequencing data. “Normalm” refers to a sample from the normal myometrium and “Normal” refers to a sample from normal cervical glands. Each number refers to the corresponding case number. Abbreviations: LEGH, lobular endocervical glandular hyperplasia; GAS, gastric-type adenocarcinoma. Supplementary Table S2. The number and percentage of each type of somatic short variant detected by whole-exome sequencing. The table shows the number and percentage of each type of short variant detected in normal cervical glands (Normal, n = 5), LEGH (n = 7), and GAS (n = 7). “All” includes all 19 samples. Abbreviations: SNV, single nucleotide variant; LEGH, lobular endocervical glandular hyperplasia; GAS, gastric-type adenocarcinoma. Supplementary Table S3. Single base substitution (SBS) mutational signature profiles. Rows represent individual samples from normal cervical glands, LEGH, and GAS. Columns show the estimated contribution of COSMIC SBS signatures (SBS1–SBS21). Values indicate the relative contribution of each signature. Zeros indicate no detectable contribution from the corresponding SBS signature. Abbreviations: SBS, single base substitutions; LEGH, lobular endocervical glandular hyperplasia; GAS, gastric-type adenocarcinoma. Supplementary Table S4. Detailed list of somatic mutations identified in representative known GAS-related genes in both LEGH and GAS samples. Variant annotation was performed using ANNOVAR. Abbreviations: LEGH, lobular endocervical glandular hyperplasia; GAS, gastric-type adenocarcinoma. Supplementary Table S5. List of genes evaluated for GAS association using the scoring system. Genes marked with an asterisk (*) were manually added for scoring based on mutation characteristics or CNA. Abbreviations: LEGH, lobular endocervical glandular hyperplasia; GAS, gastric-type adenocarcinoma; SNV, single nucleotide variant; CNA, copy number alteration; NA, not available; CN-LOH, copy-neutral loss of heterozygosity. Supplementary Table S6. List of enriched pathways. The pathways listed above the double line are statistically significant, based on Benjamini-adjusted *p*-values. Supplementary Table S7. Sensitivity analysis of the scoring threshold for candidate GAS-related genes. This table summarizes genes retained under more stringent thresholds (≥4 and ≥5) to assess threshold sensitivity
