# Supplementary material for: Identification of the Carcinogenic Process from Lobular Endocervical Glandular Hyperplasia to Gastric-Type Adenocarcinoma of the Uterine Cervix via Whole-Exome Sequencing
Source: Cancers (Basel). 2026 Feb 17;18(4):651. doi: 10.3390/cancers18040651 (PMC12939958; doi:10.3390/cancers18040651)
Supplement: Supplementary file 1 [file cancers-18-00651-s001.zip › Supplementary Figure S1.pdf]

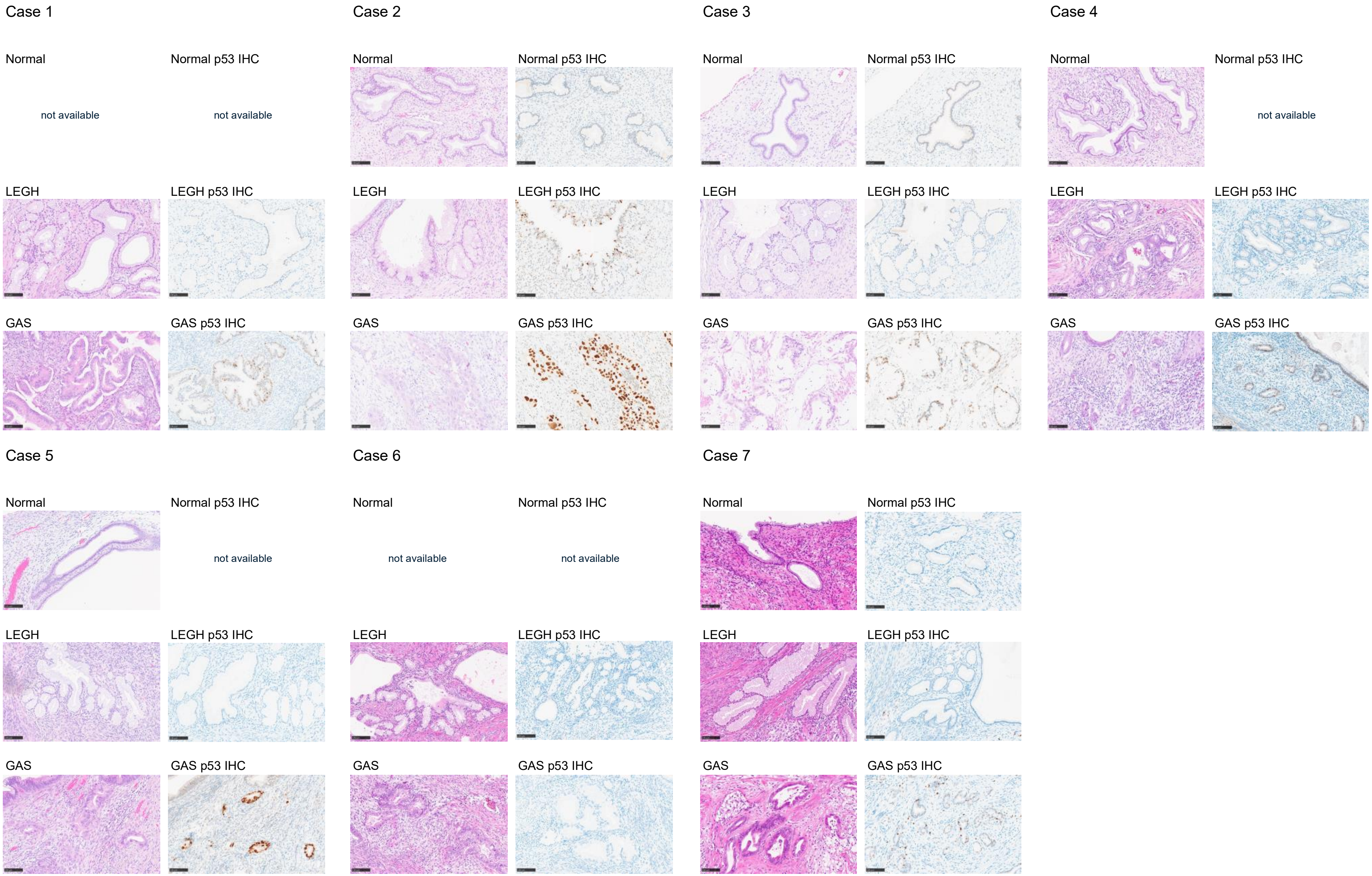
Case 1
Normal
not available
Normal p53 IHC
not available
LEGH
LEGH p53 IHC
GAS
GAS p53 IHC
Case 2
Normal
Normal p53 IHC
LEGH
LEGH p53 IHC
GAS
GAS p53 IHC
Case 3
Normal
Normal p53 IHC
LEGH
LEGH p53 IHC
GAS
GAS p53 IHC
Case 4
Normal
Normal p53 IHC
not available
LEGH
LEGH p53 IHC
GAS
GAS p53 IHC
Case 5
Normal
Normal p53 IHC
not available
LEGH
LEGH p53 IHC
GAS
GAS p53 IHC
Case 6
Normal
not available
Normal p53 IHC
not available
LEGH
LEGH p53 IHC
GAS
GAS p53 IHC
Case 7
Normal
Normal p53 IHC
LEGH
LEGH p53 IHC
GAS
GAS p53 IHC
