# Supplementary material for: Identification of the Carcinogenic Process from Lobular Endocervical Glandular Hyperplasia to Gastric-Type Adenocarcinoma of the Uterine Cervix via Whole-Exome Sequencing
Source: Cancers (Basel). 2026 Feb 17;18(4):651. doi: 10.3390/cancers18040651 (PMC12939958; doi:10.3390/cancers18040651)

A

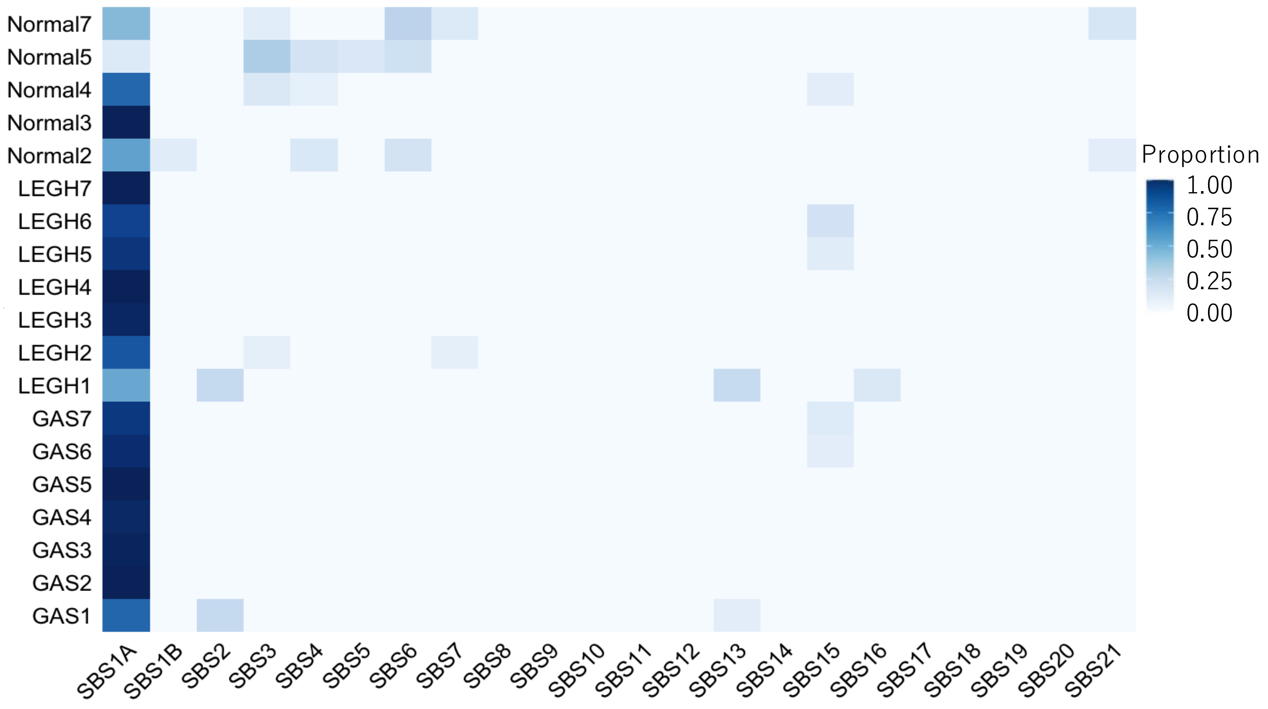
Normal7
Normal5
Normal4
Normal3
Normal2
LEGH7
LEGH6
LEGH5
LEGH4
LEGH3
LEGH2
LEGH1
GAS7
GAS6
GAS5
GAS4
GAS3
GAS2
GAS1
SBS1A
SBS1B
SBS2
SBS3
SBS4
SBS5
SBS6
SBS7
SBS8
SBS9
SBS10
SBS11
SBS12
SBS13
SBS14
SBS15
SBS16
SBS17
SBS18
SBS19
SBS20
SBS21
Proportion
1.00
0.75
0.50
0.25
0.00

B

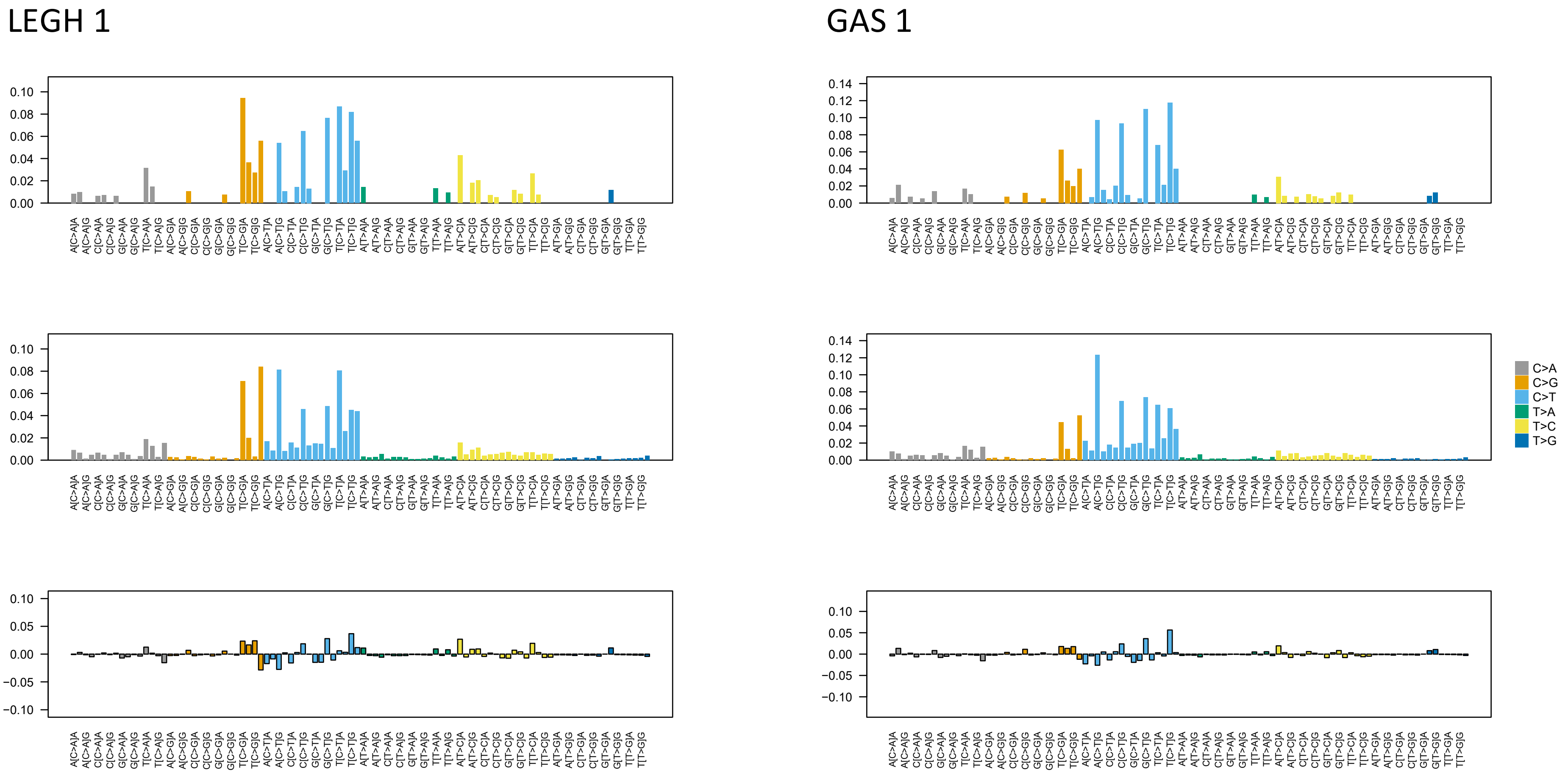

C

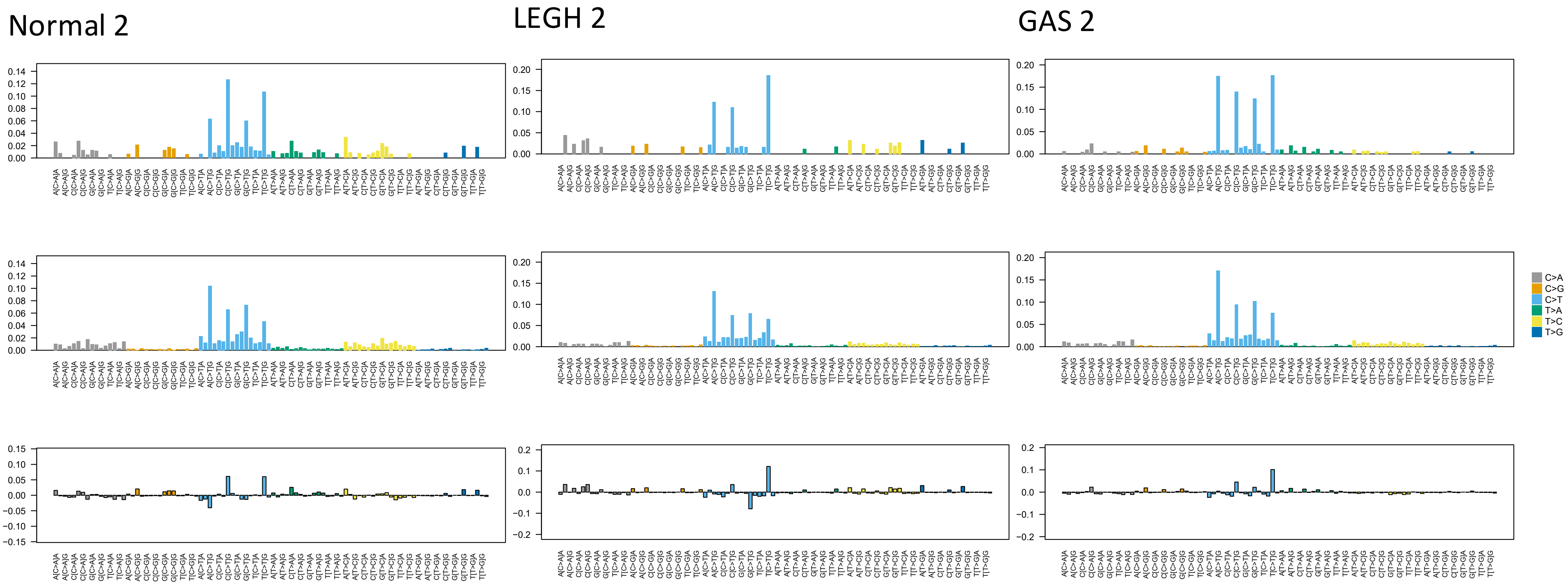

D

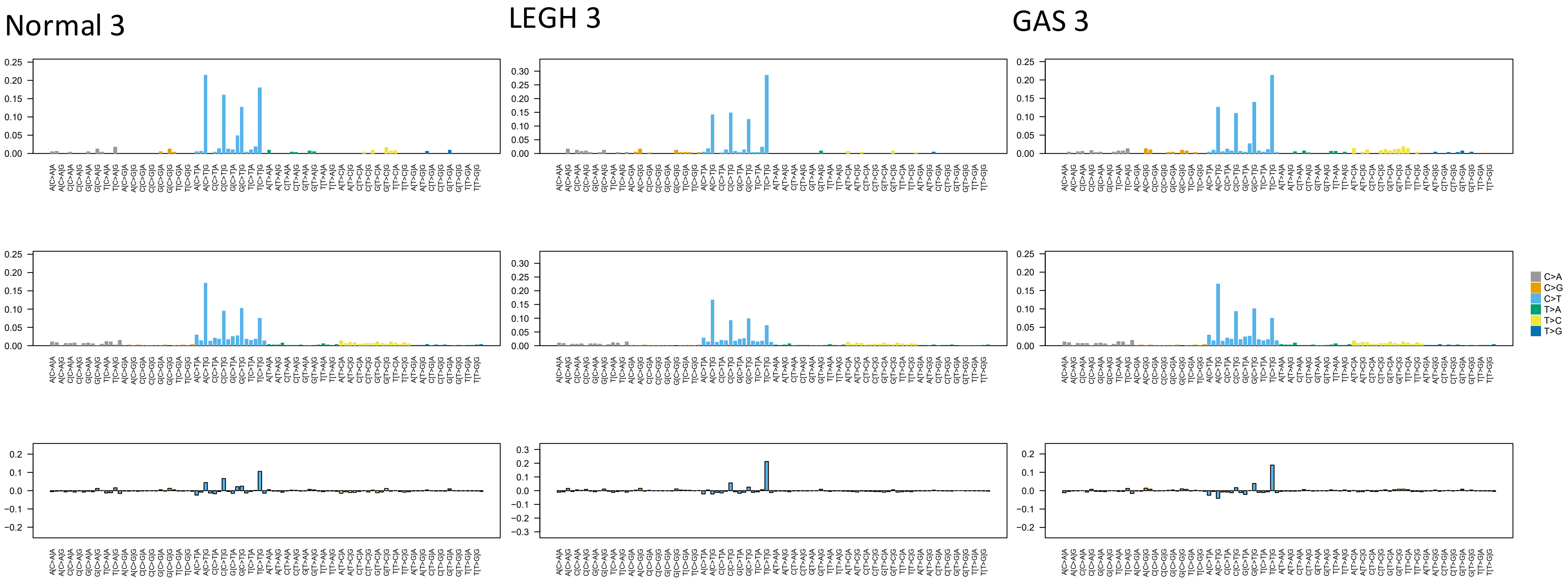

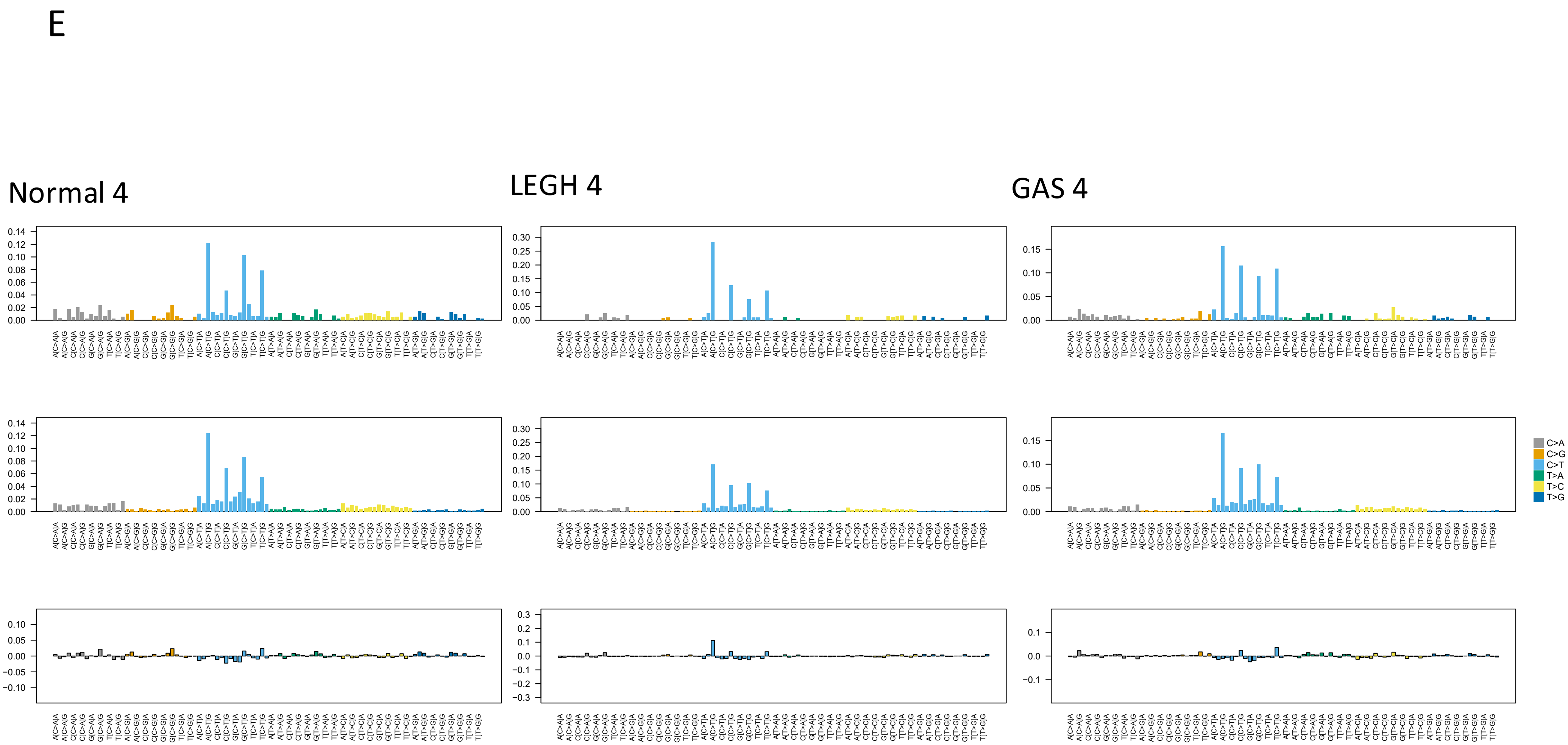
E
Normal 4
LEGH 4
GAS 4
C>A
C>G
C>T
T>A
T>C
T>G

F

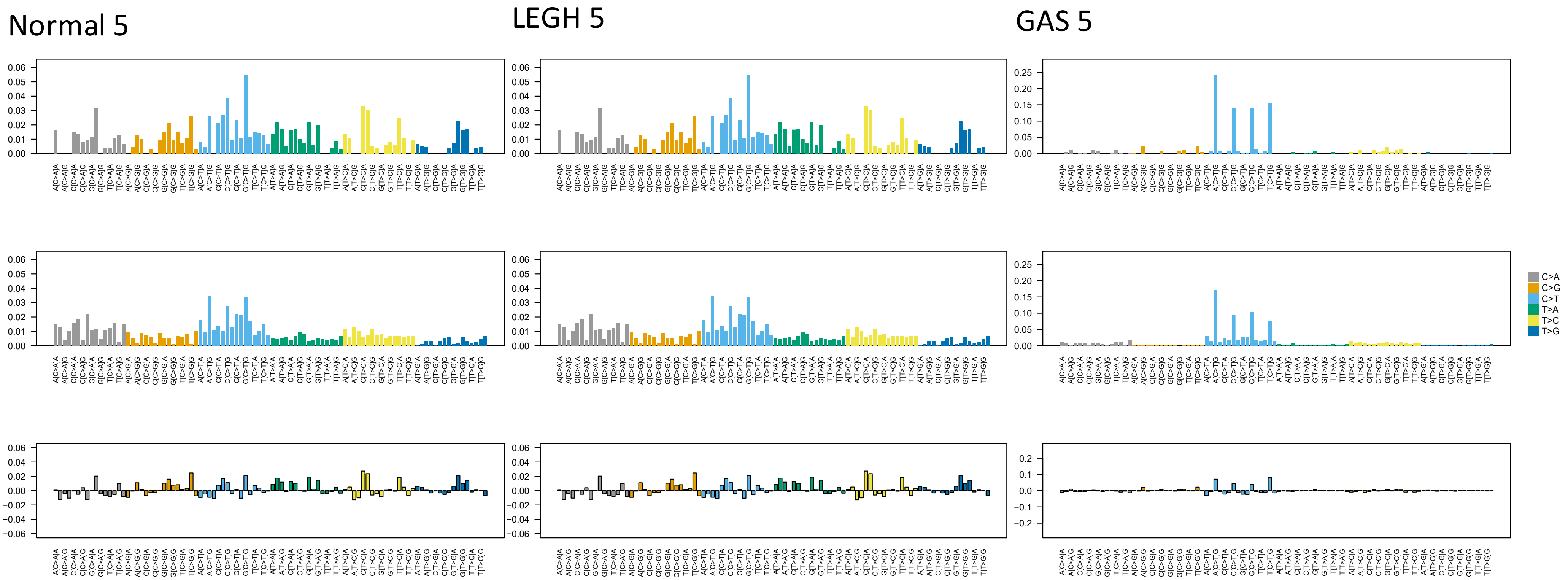

G

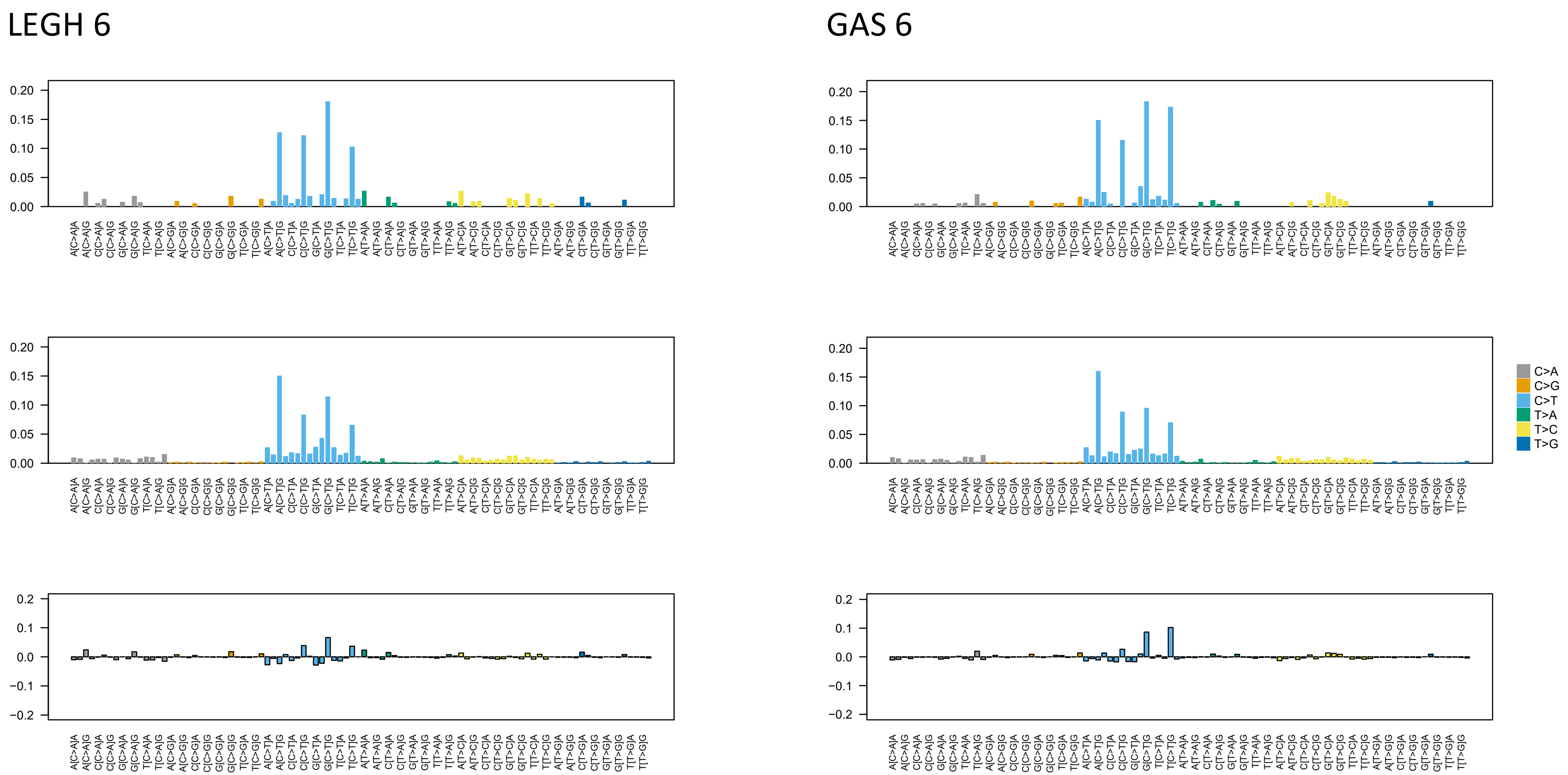

H

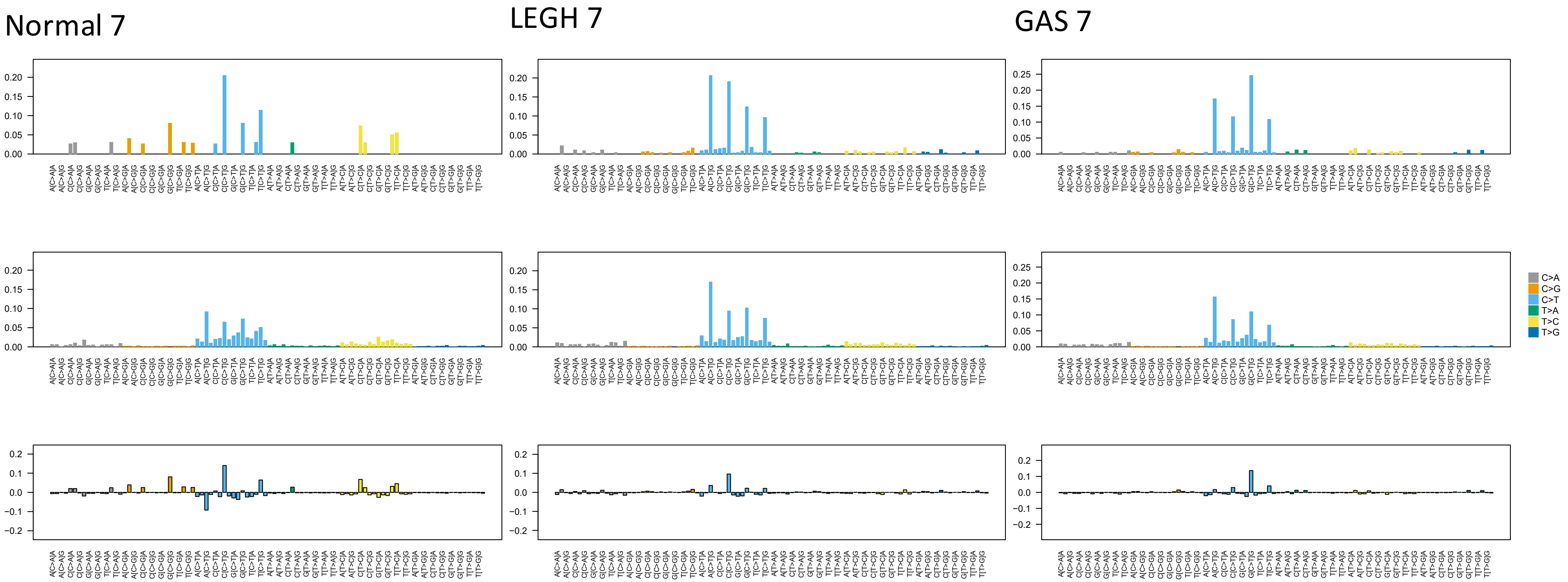

Supplement: Supplementary file 1 [file cancers-18-00651-s001.zip › Supplementary Figure S3.pdf]
