# Supplementary material for: Identification of the Carcinogenic Process from Lobular Endocervical Glandular Hyperplasia to Gastric-Type Adenocarcinoma of the Uterine Cervix via Whole-Exome Sequencing
Source: Cancers (Basel). 2026 Feb 17;18(4):651. doi: 10.3390/cancers18040651 (PMC12939958; doi:10.3390/cancers18040651)
Supplement: Supplementary file 1 [file cancers-18-00651-s001.zip › Supplementary Figure S4.pdf]

A

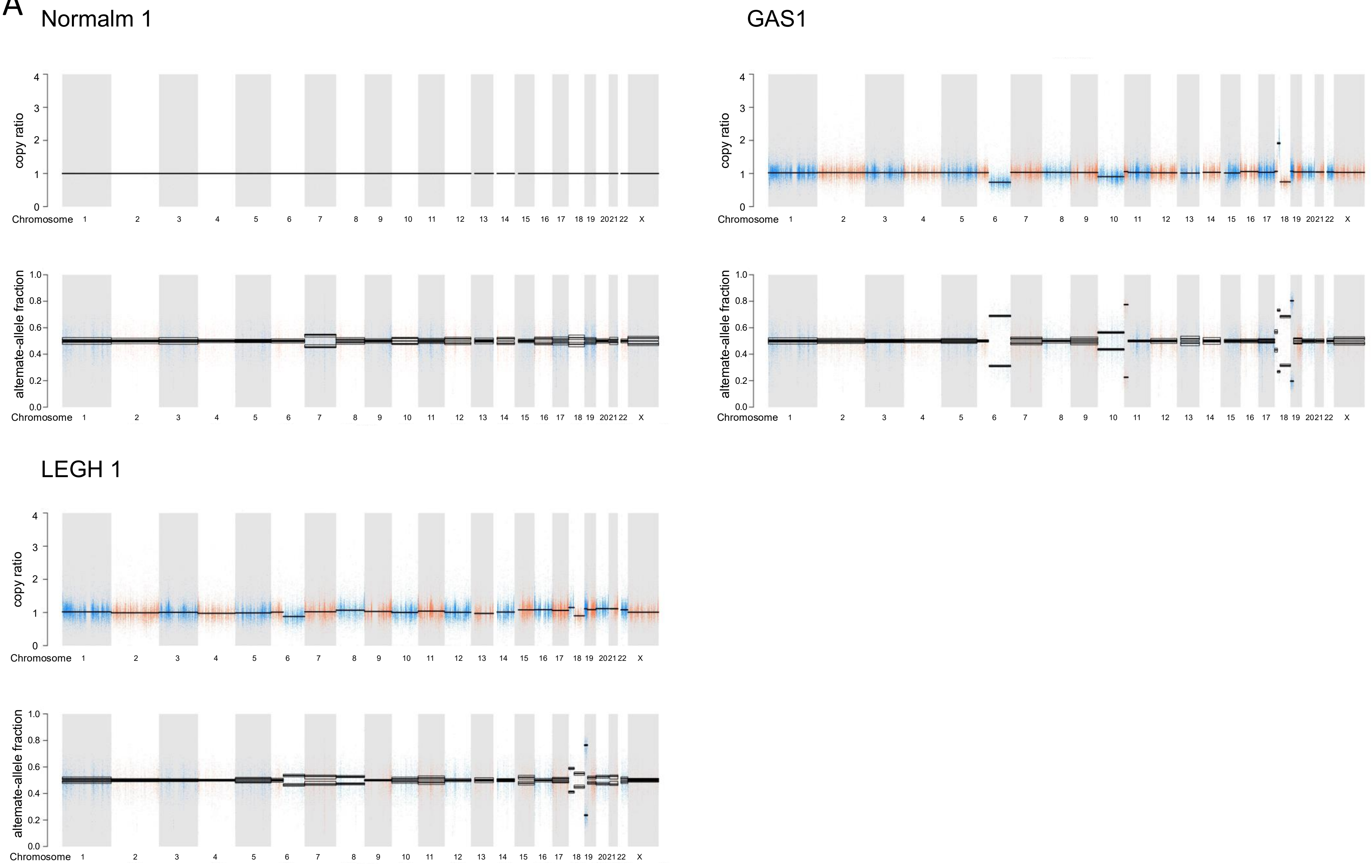
Normalm 1
GAS1
LEGH 1
copy ratio
alternate-allele fraction
Chromosome
1 2 3 4 5 6 7 8 9 10 11 12 13 14 15 16 17 18 19 20 21 22 X

B

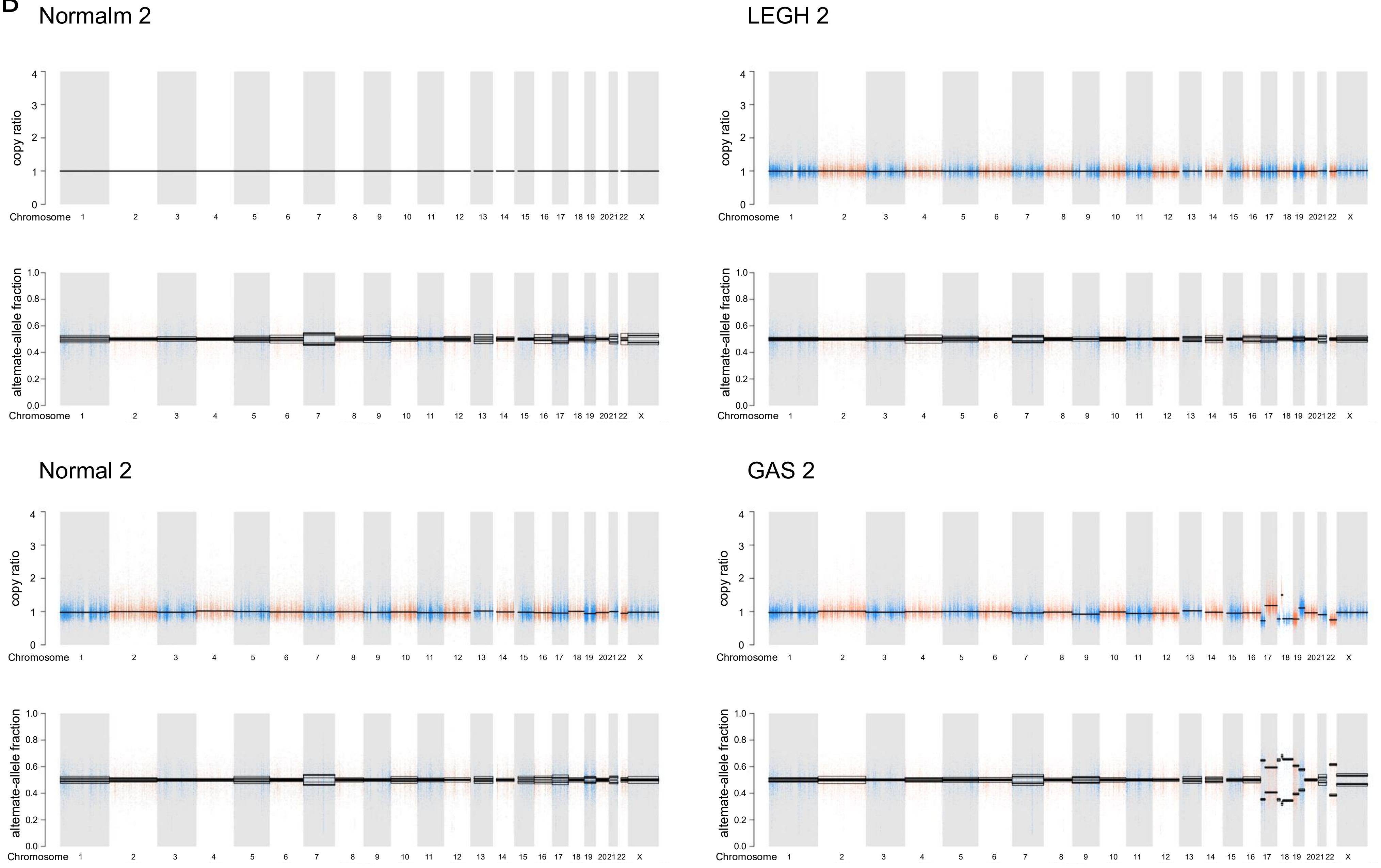

Normalm 2
LEGH 2
Normal 2
GAS 2
copy ratio
alternate-allele fraction
Chromosome
1 2 3 4 5 6 7 8 9 10 11 12 13 14 15 16 17 18 19 20 21 22 X

C

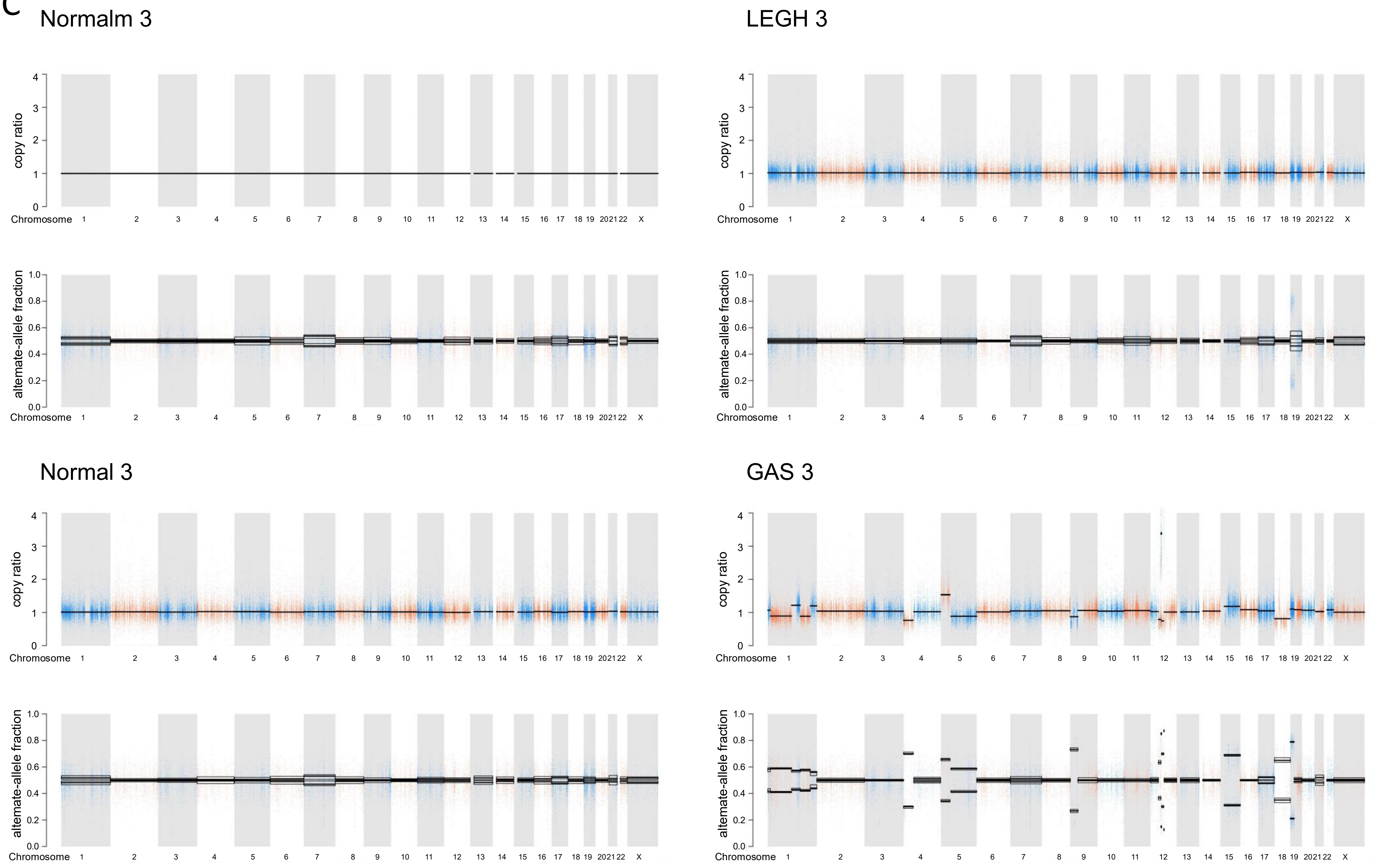

Normalm 3
LEGH 3
Normal 3
GAS 3
copy ratio
alternate-allele fraction
Chromosome
1 2 3 4 5 6 7 8 9 10 11 12 13 14 15 16 17 18 19 20 21 22 X

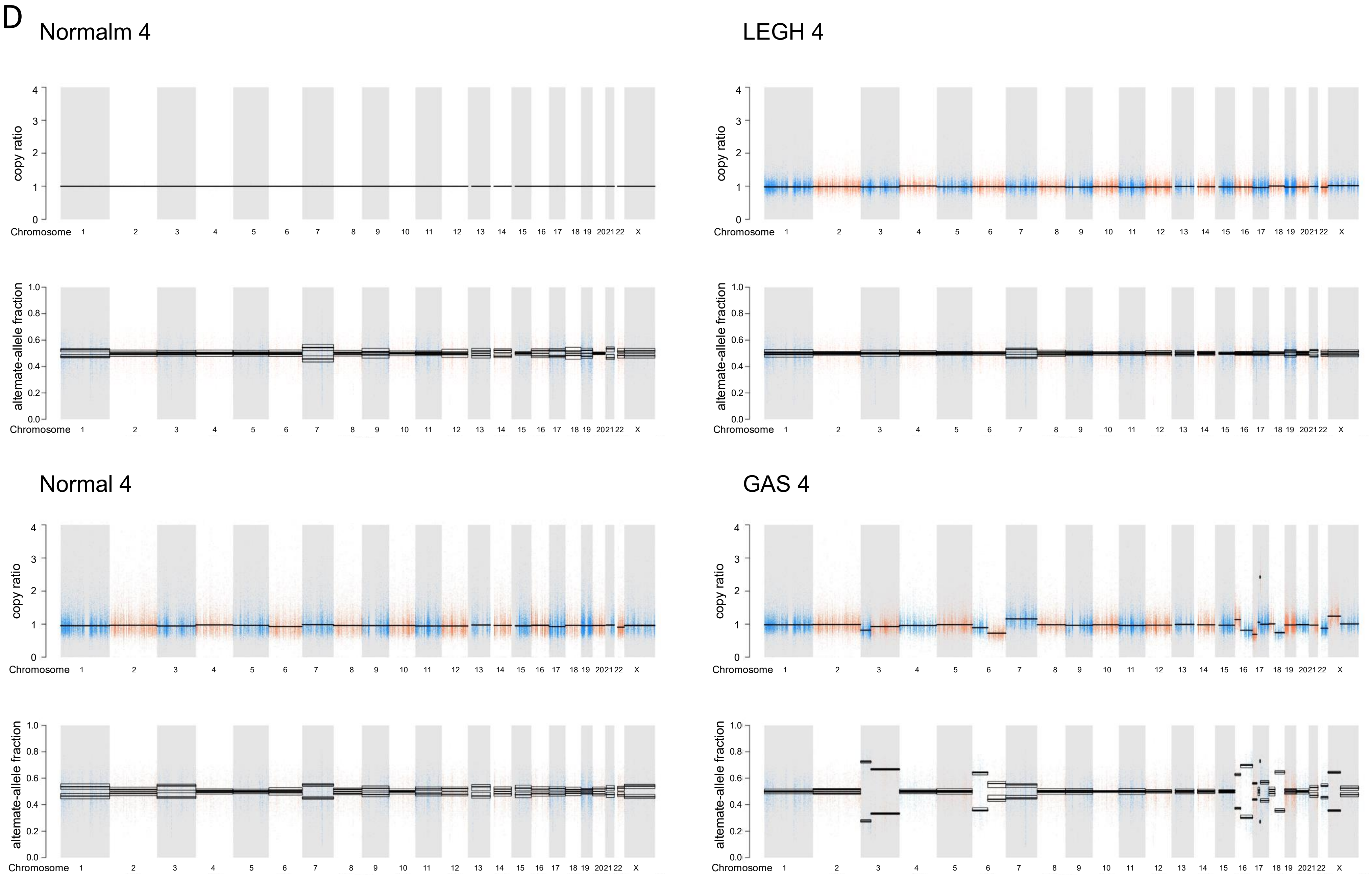

D
Normalm 4
LEGH 4
Normal 4
GAS 4
copy ratio
altemate-allele fraction
Chromosome
1 2 3 4 5 6 7 8 9 10 11 12 13 14 15 16 17 18 19 2021 22 X

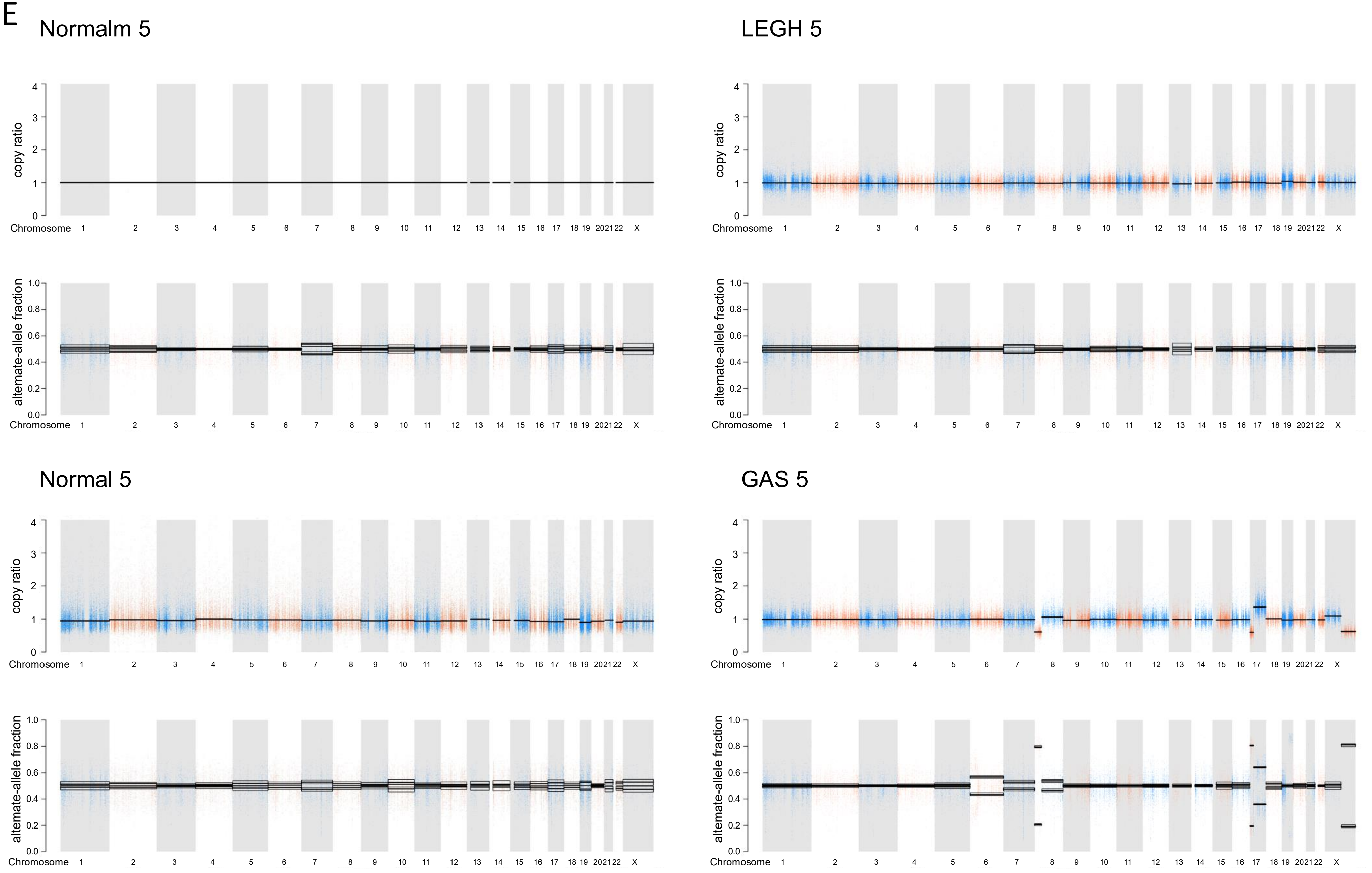
E
Normalm 5
LEGH 5
Normal 5
GAS 5
copy ratio
altemate-allele fraction
Chromosome
1 2 3 4 5 6 7 8 9 10 11 12 13 14 15 16 17 18 19 20 21 22 X

F

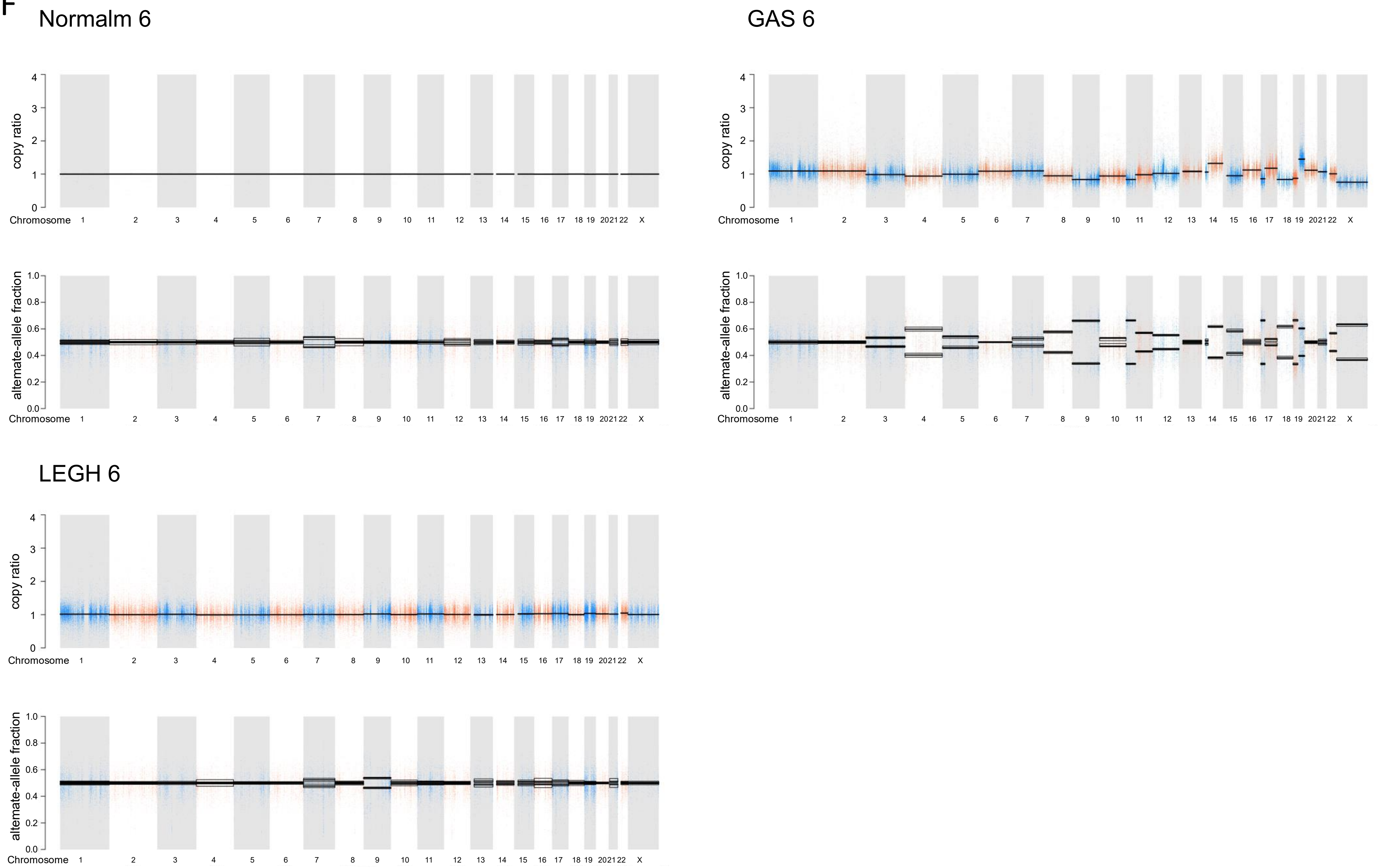
Normalm 6
GAS 6
LEGH 6
copy ratio
alternate-allele fraction
Chromosome
1 2 3 4 5 6 7 8 9 10 11 12 13 14 15 16 17 18 19 20 21 22 X

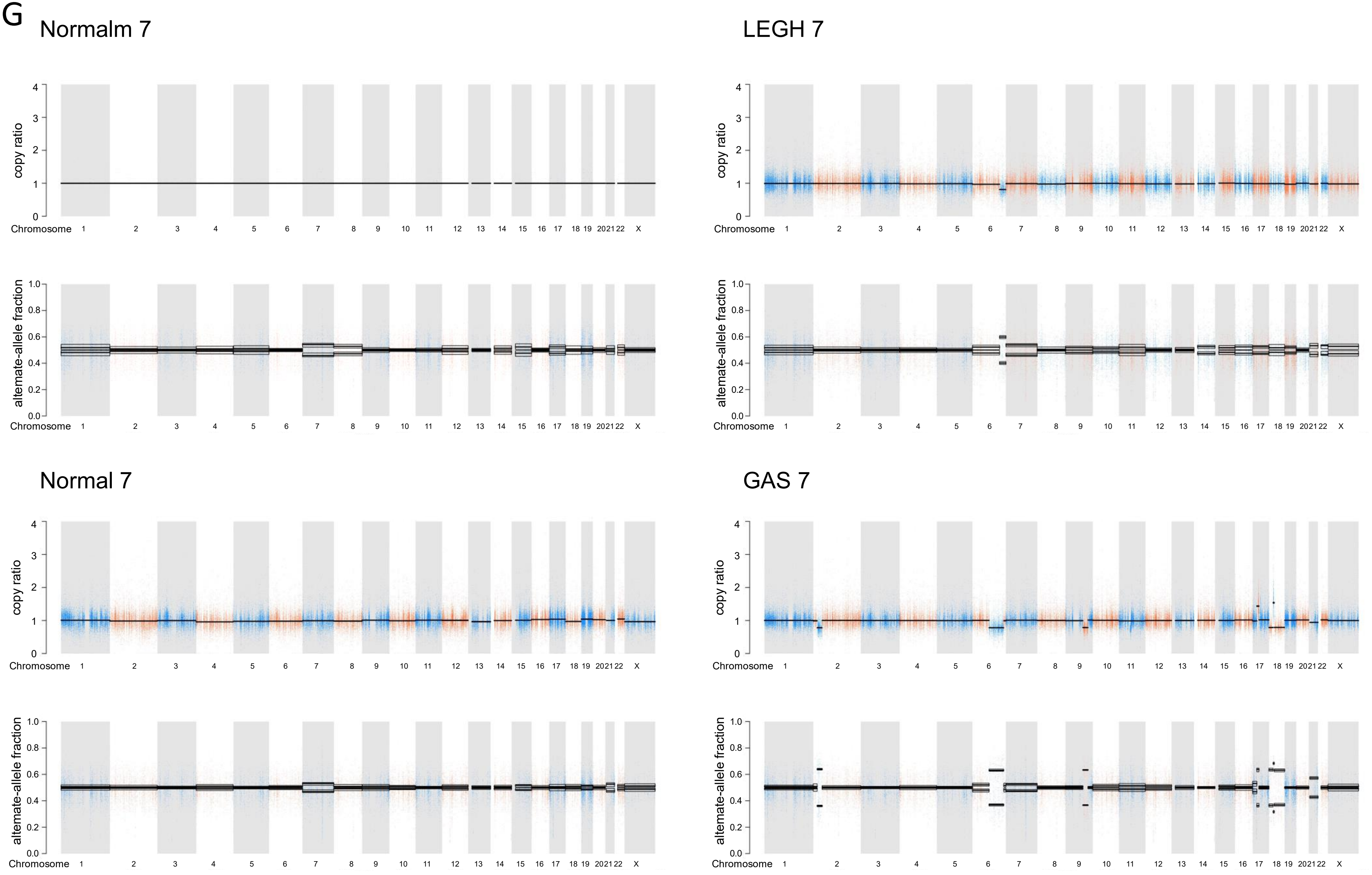

G
Normalm 7
LEGH 7
Normal 7
GAS 7
copy ratio
alternate-allele fraction
Chromosome
1 2 3 4 5 6 7 8 9 10 11 12 13 14 15 16 17 18 19 20 21 22 X
