# Supplementary material for: Identification of the Carcinogenic Process from Lobular Endocervical Glandular Hyperplasia to Gastric-Type Adenocarcinoma of the Uterine Cervix via Whole-Exome Sequencing
Source: Cancers (Basel). 2026 Feb 17;18(4):651. doi: 10.3390/cancers18040651 (PMC12939958; doi:10.3390/cancers18040651)

A

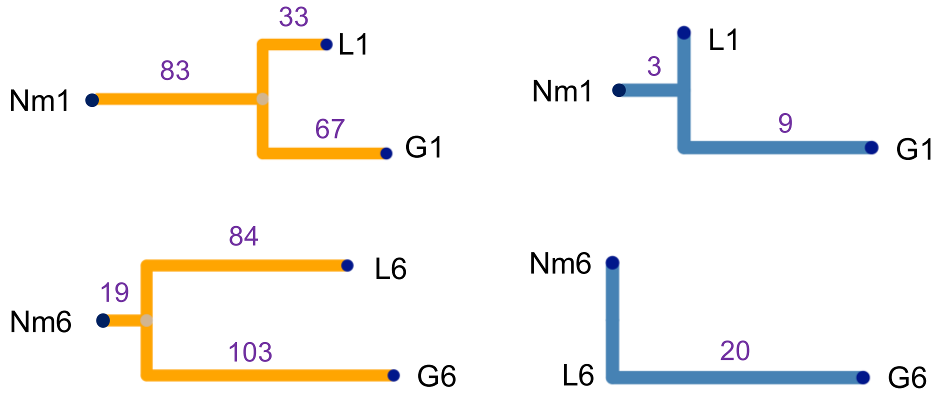

B

Case	Newick format for SNV
1	(LEGH1:0.15578,Normal_m1:0.40704,GAS1:0.30151);
2	((Normal2:0.35479,LEGH2:0.11370):0.01918,Normalm2:0.01781,GAS2:0.31096);
3	(Normal3:0.27561,(LEGH3:0.26098,GAS3:0.35122):0.03171,Normalm3:0.02195);
4	(LEGH4:0.12451,(Normal4:0.43774,Normalm4:0.00000):0.00778,GAS4:0.36187);
5	(LEGH5:0.21753,(Normal5:0.37500,Normalm5:0.00000):0.02922,GAS5:0.28896);
6	(LEGH6:0.34211,GAS6:0.42105,Normalm6:0.07895);
7	(LEGH7:0.49640,(Normal7:0.08273,GAS7:0.32374):0.02878,Normalm7:0.00000);

C

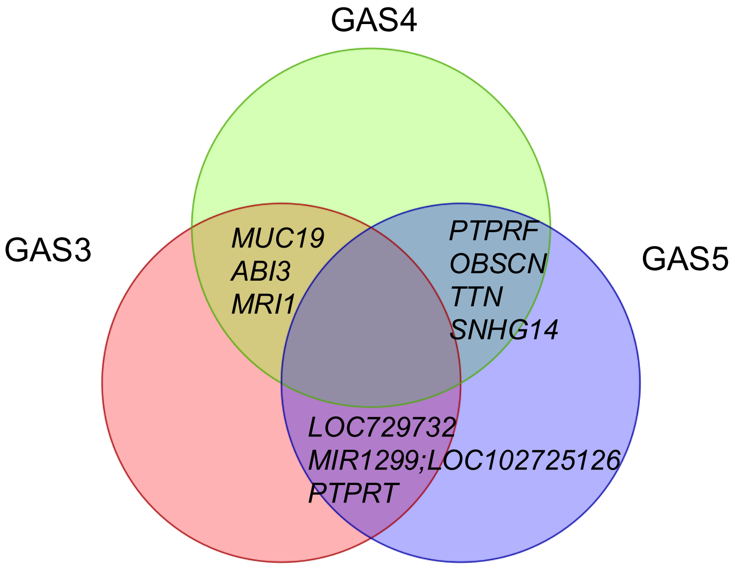

Supplement: Supplementary file 1 [file cancers-18-00651-s001.zip › Supplementary Figure S6.pdf]
