# Supplementary material for: Identification of the Carcinogenic Process from Lobular Endocervical Glandular Hyperplasia to Gastric-Type Adenocarcinoma of the Uterine Cervix via Whole-Exome Sequencing
Source: Cancers (Basel). 2026 Feb 17;18(4):651. doi: 10.3390/cancers18040651 (PMC12939958; doi:10.3390/cancers18040651)
Supplement: Supplementary file 1 [file cancers-18-00651-s001.zip › Supplementary Table S2.pdf]

n (%)

Type of short variants	All (19 samples)	Normal (5 samples)	LEGH (7 samples)	GAS (7 samples)
Nonsynomymous SNV	624 (58.9)	225 (63.2)	150 (55.6)	249 (57.5)
Frameshift indel	43 (4.1)	16 (4.5)	5 (1.9)	22 (5.1)
Stopgain	35 (3.3)	9 (2.5)	9 (3.3)	17 (3.9)
Stoploss	1 (0.1)	0 (0.0)	1 (0.4)	0 (0.0)
Nonframeshift indel	24 (2.3)	9 (2.5)	6 (2.2)	9 (2.1)
Synonymous SNV	312 (29.5)	92 (25.8)	92 (34.1)	128 (29.6)
Others	20 (1.9)	5 (1.4)	7 (2.6)	8 (1.8)
