# Supplementary material for: Identification of the Carcinogenic Process from Lobular Endocervical Glandular Hyperplasia to Gastric-Type Adenocarcinoma of the Uterine Cervix via Whole-Exome Sequencing
Source: Cancers (Basel). 2026 Feb 17;18(4):651. doi: 10.3390/cancers18040651 (PMC12939958; doi:10.3390/cancers18040651)
Supplement: Supplementary file 1 [file cancers-18-00651-s001.zip › Supplementary Table S7.pdf]

| Score | The number of genes | Gene |
|---|---|---|
| ≧4 | 13 | *SMAD4, TP53, ERBB2, SMAD2, ARID1B, MAP2K4, YWHAE, PER1, NCOR1, FLCN, SUZ12, IKZF3, CDKN2A* |
| ≧5 | 6 | *SMAD4, TP53, ERBB2, SMAD2, ARID1B, MAP2K4* |
