# Supplementary figures and images for: Identification of the Carcinogenic Process from Lobular Endocervical Glandular Hyperplasia to Gastric-Type Adenocarcinoma of the Uterine Cervix via Whole-Exome Sequencing
Source: Cancers (Basel). 2026 Feb 17;18(4):651. doi: 10.3390/cancers18040651 (PMC12939958; doi:10.3390/cancers18040651)

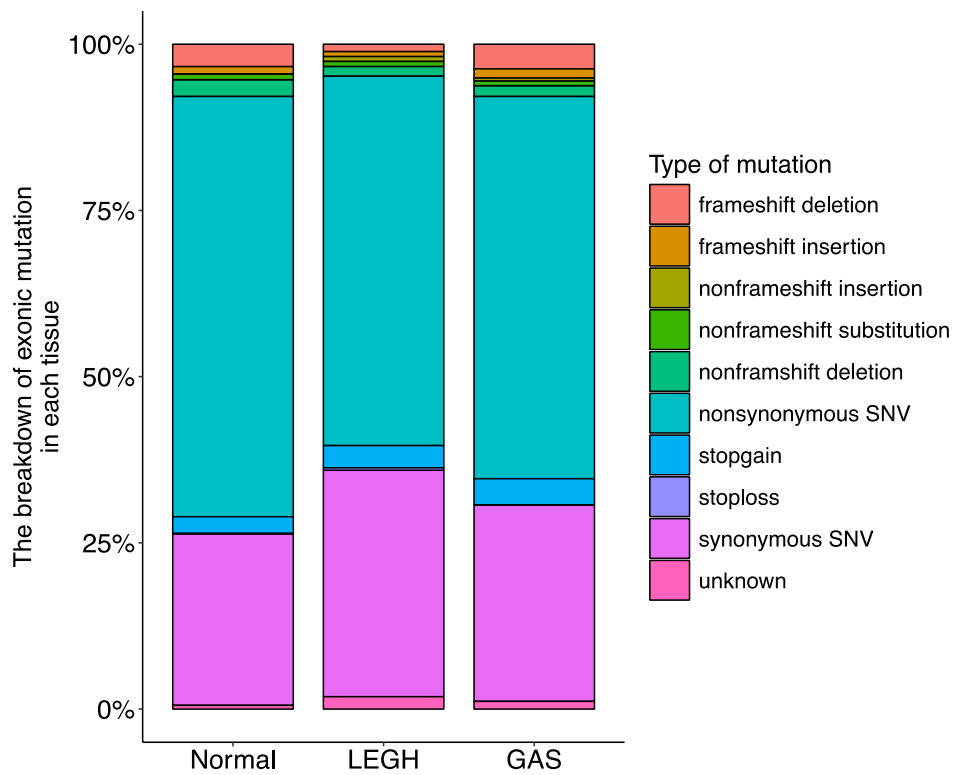

Supplement: Supplementary file 1 [file cancers-18-00651-s001.zip › Supplementary Figure S2.pdf]

A Case 1

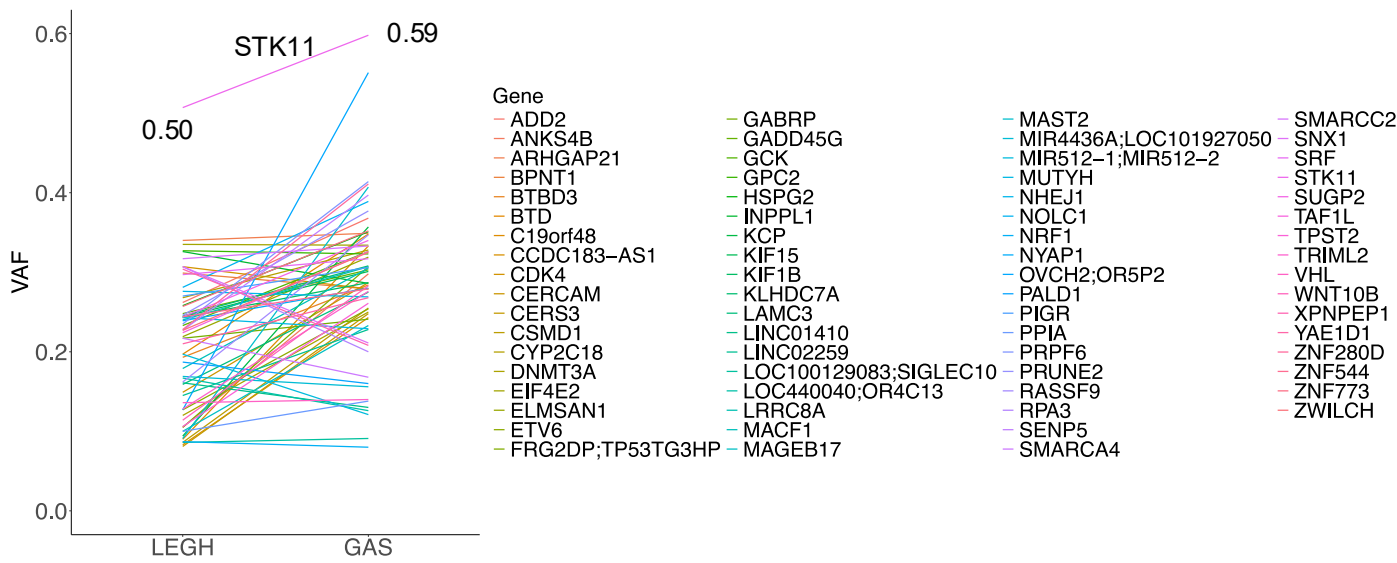

B Case 2

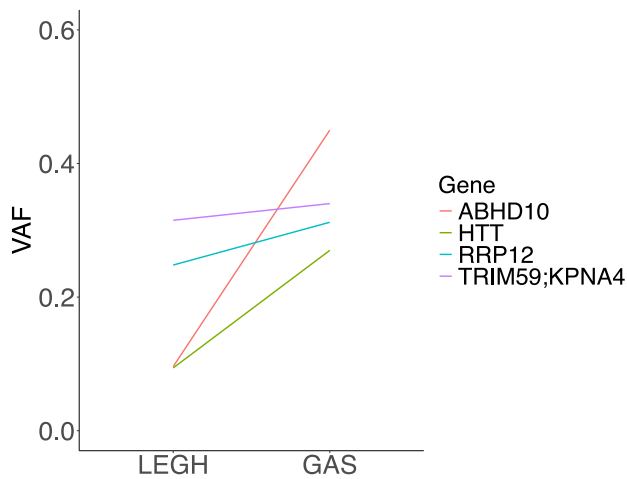

C Case 3

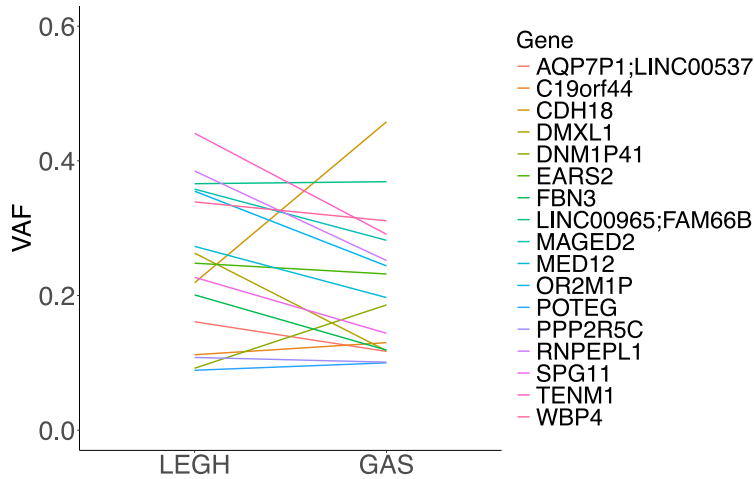

D Case 4

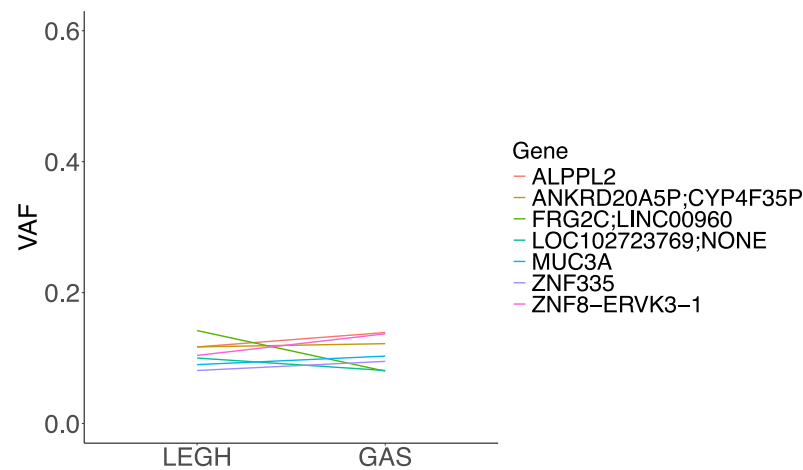

E Case 5

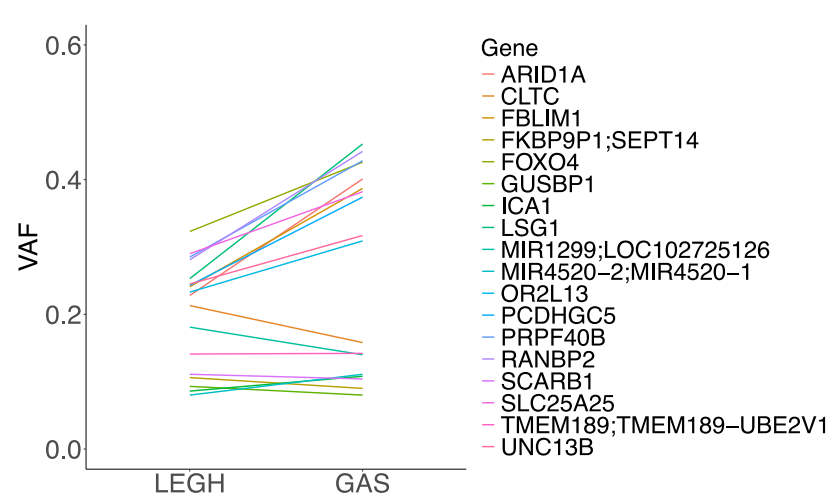

F Case 6

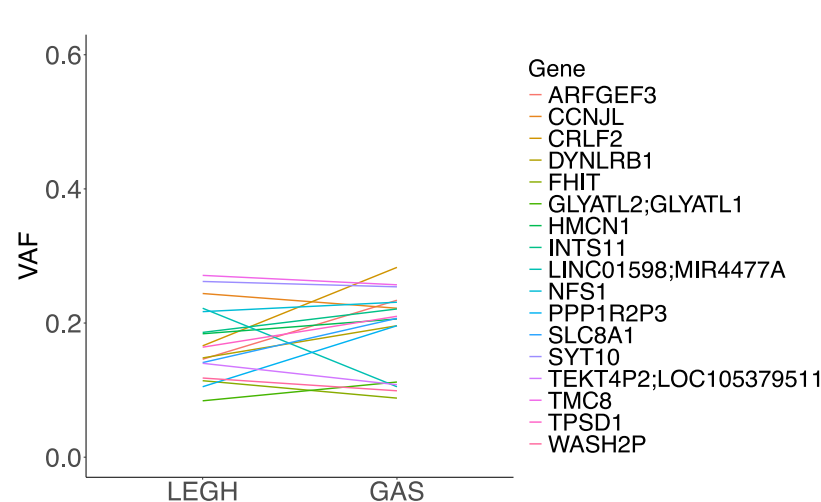

Supplement: Supplementary file 1 [file cancers-18-00651-s001.zip › Supplementary Figure S5 .pdf]
